# Multiple *Streptomyces* species with distinct secondary metabolomes have identical 16S rRNA gene sequences

**DOI:** 10.1038/s41598-017-11363-1

**Published:** 2017-09-11

**Authors:** Sanjay Antony-Babu, Didier Stien, Véronique Eparvier, Delphine Parrot, Sophie Tomasi, Marcelino T. Suzuki

**Affiliations:** 1 Sorbonne Universités, UPMC Univ Paris 06, CNRS, Laboratoire de Biodiversité et Biotechnologies Microbiennes (LBBM), Observatoire Océanologique, F-66650 Banyuls/Mer, France CNRS, USR 3579, LBBM, Observatoire Océanologique, 66650 Banyuls-sur-Mer, France; 2 CNRS, Institut de Chimie des Substances Naturelles, 91198 Gif-sur-Yvette cedex, France; 3 UMR CNRS 6226, Institut des Sciences Chimiques de Rennes, Equipe CORINT “Chimie Organique et Interface”, UFR Sciences Pharmaceutiques et Biologiques, Univ. Rennes 1, Université Bretagne Loire, 2 Avenue du Pr. Léon Bernard, F-35043 Rennes, France; 4 Present Address: School of Biological Sciences, University of Nebraska-Lincoln, Lincoln, Nebraska 68588 United States of America

## Abstract

Microbial diversity studies using small subunit (SSU) rRNA gene sequences continue to advance our understanding of biological and ecological systems. Although a good predictor of overall diversity, using this gene to infer the presence of a species in a sample is more controversial. Here, we present a detailed polyphasic analysis of 10 bacterial strains isolated from three coastal lichens *Lichina confinis, Lichina pygmaea* and *Roccella fuciformis* with SSU rRNA gene sequences identical to the type strain of *Streptomyces cyaneofuscatus*. This analysis included phenotypic, microscopic, genetic and genomic comparisons and showed that despite their identical SSU rRNA sequences the strains had markedly different properties, and could be distinguished as 5 different species. Significantly, secondary metabolites profiles from these strains were also found to be different. It is thus clear that SSU rRNA based operational taxonomy units, even at the most stringent cut-off can represent multiple bacterial species, and that at least for the case of *Streptomyces*, strain de-replication based on SSU gene sequences prior to screening for bioactive molecules can miss potentially interesting novel molecules produced by this group that is notorious for the production of drug-leads.

## Introduction

It is now clear that almost all eukaryotic organisms have an associated prokaryotic microflora. Thanks to advances in massively parallel sequencing technology, accessibility to high performance computing (HPC), and the development of comparative analytical software, the number of prokaryotic microflora surveys have increased in recent years^1^. These studies are mostly based on the analysis of small subunit (SSU) rRNA diversity^2^ and take advantage of the correlation between the gene sequence and prokaryotic taxonomical hierarchy^3^. In this type of studies sequences are clustered together at a certain level of identity (97% in the vast majority of the cases) in what are termed Operational Taxonomic Units (OTUs)^4^. While such OTU-based analysis is very useful for describing major patterns of microbial diversity, they are increasingly being used as proxies to bacterial species^5^. It is also common to find ecological or physiological traits attributed to OTUs of uncultured microorganisms based on similarities of their SSU rRNA gene sequences to those of bacterial strains with known *ecological or physiological* traits^6^. However, it is not clear whether these OTUs are functionally coherent, that is, whether *strains assigned to a single OTU represent a single species* and whether *strains with identical SSU rRNA gene sequences exhibit similar ecological or physiological functions*.

To answer the above-mentioned questions, one approach would be to study a bacterial taxon that is widespread across different ecosystems and niches, potentially represents keystone organisms ﻿and that are metabolically active. Members of the genus *Streptomyces* represent good model organisms with such properties^7–11^ and well-known for their ability to produce vast numbers of biologically-active secondary metabolites^12^. Since these molecules play central roles in microbial competition and inter- and intra- species signaling^13^ they represent a phenotypic characteristic with niche determining potential^14^, and due to their bioactivity have extensively been developed as drugs^12^. To add further significance to *Streptomyces* as model for the current study, systematics of these bacteria have had a “turbulent” past^15^ where close to 3000 named-species existed the 1970s albeit, almost solely on patent literature^16^. After several efforts to amend their taxonomy^17^, around 600 species of *Streptomyces* are currently officially recognized^18^ and those are classified in 130 supra-specific clades^17^. SSU rRNA gene-sequencing have played a pivotal role in solving many discrepancies in their taxonomy^17, 19, 20^, regardless of the diversity observed among strains with similar SSU rRNA gene sequences^20–24^.

In the study presented here, the phenotypes of 10 *Streptomyces* strains, isolated as part of an earlier microbial diversity study^25^ from three different lichens (*Lichina confinis, Lichina pygmeae* and *Roccella fuciformis*), using different treatments, and that had 100% similar SSU rRNA gene sequences were compared. The strains were subject of morphological, phenotypical, molecular taxonomic and metabolic analyses to test whether stains with identical SSU rRNA gene sequences represented the same species, and presented similar functional traits.

## Results

### Stress resistance phenotypic assays

Stress resistance was evaluated from as increase in dry weight after growth in International Streptomyces Project media 2 (ISP2) that had been either amended with NaCl (5, 10 and 20%) or adjusted to the different pH levels (5.0, 7.0 & 9.0; Table 1). The individual growth curves are presented in supplementary Figure S1.

**Table 1:** Source of the 10 selected strains from Parrot *et al*. (2015) used in this study, and their phenotypic and genomic characteristics.

Isolate code	Lichen source	Medium of isolation^a^	Inoculum type^b^	Halotolerance – NaCl %			Growth at different pH			Color group number	GC %
				5%	10%	20%	5	7	9		
MOLA1420	*Lichina pygmaea*	MA	H		++			++	+	1	64%
MOLA1421		MA	H		++			++	+	1	64%
MOLA1427		AIA	H		++			++		2	64%
MOLA1488	*Lichina confinis*	AIA	W	+	++			++	+	3	64%
MOLA1493		AIA	W	+	++			++	++	4	66%
MOLA1578	*Roccella fuciformis*	AIA	H	++	++			++	++	5	66%
MOLA1596		AIA	W	++	++			++	++	5	66%
MOLA1615		AIA	W	++	++			++	++	5	66%
MOLA1611		AIA	W	++	++	+	+	++	++	6	64%
MOLA1612		AIA	W	++	++	+	+	++	++	6	64%

^a^MA- marine agar, AIA – actinomycetes isolation agar.

^b^H- homogenate, W- Wash.

#### Halotolerance

All 10 tested strains grew well in 10% NaCl and the strains could be clustered into 4 groups based on the growth kinetics at different concentrations of NaCl (Table 1). Three strains, MOLA1420, MOLA1421 and MOLA1427, all isolated from *Lichina pygmaea*, only grew with 10% NaCl. Interestingly, although MOLA1420 and MOLA1421 were isolated on marine agar they were not particularly more halotolerant when compared to some strains isolated using actinomycetes isolation agar. MOLA1488 and MOLA1493, both strains that originated from *Lichina confinis*, showed moderate growth at 5% salt (in addition to optimal growth at 10%). Five strains isolated from *Roccella fuciformis* could be separated into two groups based on their growth at different salt concentrations and all five strains grew well in 5% and 10% salts. MOLA1578, MOLA1596 and MOLA1615 could not grow at 20% that differentiated them from the MOLA1611 and MOLA1612, which could.

#### Optimal growth pH

All test strains grew well in pH 7.0. Based on the growth kinetics at different pHs, the strains could be clustered into 4 groups. Unlike with their salt tolerance, pH growth kinetics had little correlation with the lichens of origin. Particularly two groups could be defined in isolates from different lichens: MOLA1420 and MOLA1421 that were isolated from *Lichina pygmaea* and MOLA1488 from *L. confinis* showed trace growth at pH 9.0 along with optimal growth at pH 7.0; MOLA1493 from *L. confinis* and MOLA1578, MOLA1596 and MOLA1615 from *R. fuciformis* showed good growth in both pH 7.0 and 9.0, but no growth at 5.0. MOLA1611 and MOLA1612 from *R. fuciformis* had the widest growth range with trace growth at pH 5.0 and good growth at pH 7.0 and 9.0. Such wide pH range is typical of alkaliphilic members of the genus *Streptomyces* ^26^. MOLA1427 from *L. pygmaea* was the only strain that grew solely at pH 7.0 and was the most stringent in terms of pH requirement.

### Color groups and morphology

**Figure 1:**
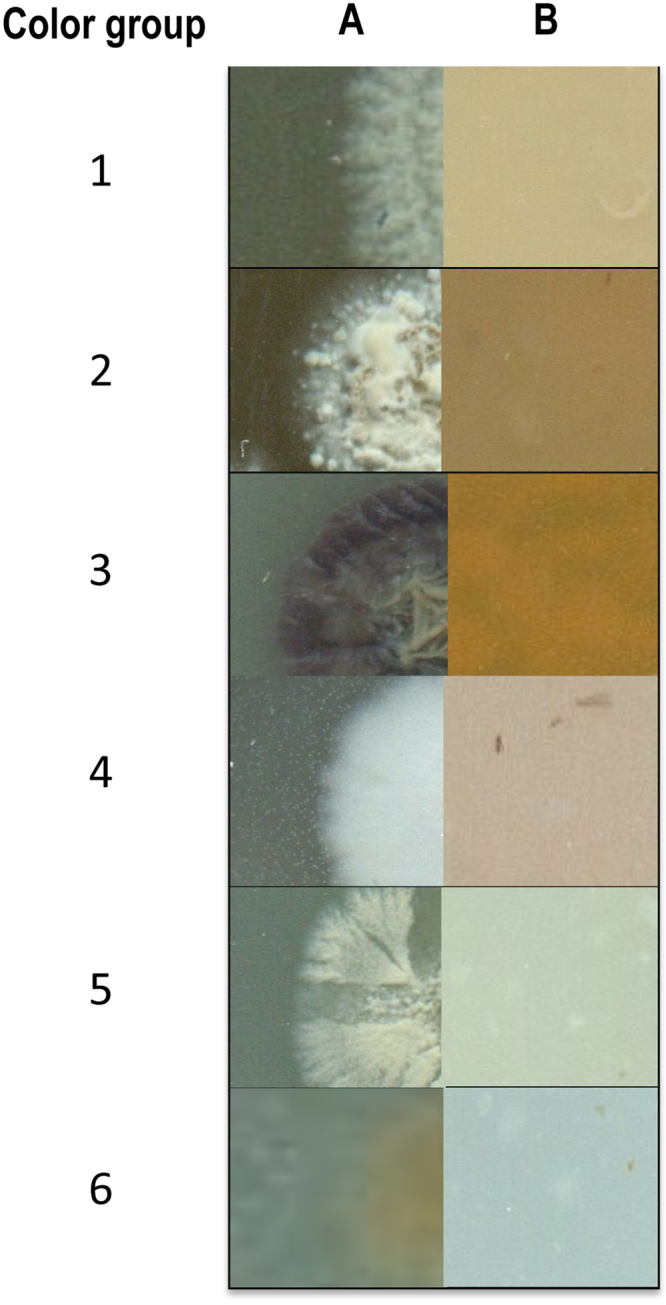
Colony morphology on oatmeal agar (ISP 3) plates of each of the color group recorded from the 10 test strains. (**A**) Edge of colony showing aerial spore mass & diffusible pigments, and (**B**) Colony reverse.

Color group properties are listed in supplementary Table S1 and the numbers are correlated with the isolates in Table 1. A total of 6 color groups were found, with 3 color groups represented by multiple strains and 3 represented by a single strain. Figure 1 shows the colony characteristics and Figure S2 spore chain morphology respectively, that were associated to the 6 different color groups. All strains isolated from *Lichina* spp. could produce melanin on ISP 6 while the 5 strains from *Roccella fuciformis* could not. Remarkably, and in partial agreement with the halotolerance experiments, each color group included strains isolated from a single lichen species and isolates from each of the three lichens belonged to two different color groups. Except for MOLA1488, all strains produced spores on ISP 3 media. All strains from *Lichina pygmaea* in color groups 1 and 2 showed the same spore chain arrangement while the two isolates from *L. confinis* showed very different colony characteristics on ISP 3 with one of them, MOLA1488 not producing any spores whereas MOLA1496 showed spores held in clusters. The five tested isolates from *Roccella fuciformis* belonged to color groups 5 and 6, and had spores arranged in chains. Members of color group 5 have spirally arranged spores in contrast to the straight chains observed in group 6.

### Protein coding genes

**Figure 2:**
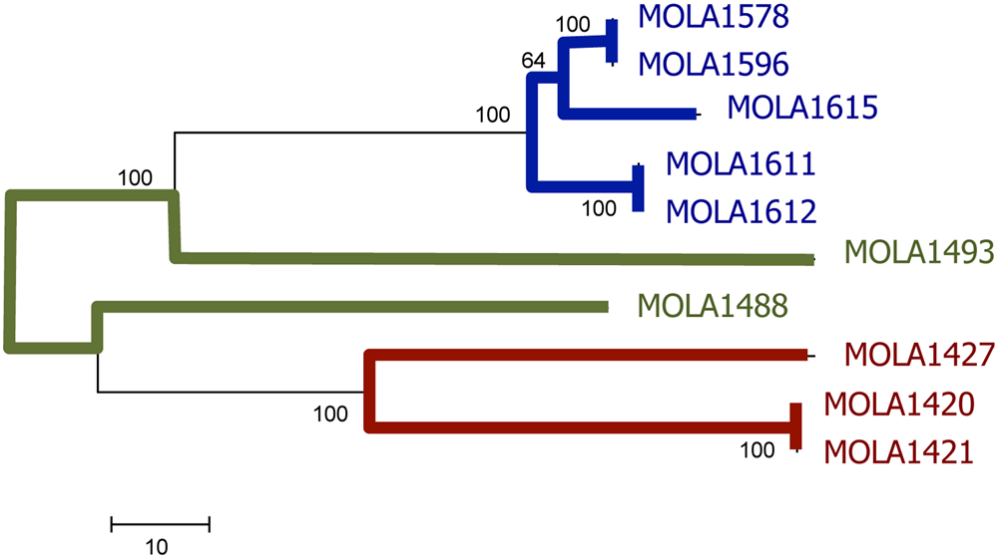
Tree showing diversity between the strains with identical SSU rRNA gene sequences. Branch colors represent the lichen sources as follows: blue *R. fuciformis*, green *L. confinis,* red *L. pygmaea*. Phylogenetic tree inferred on concatenated sequences of the 5 protein coding sequences (atpD, gyrB, recA, rpoB and trpD) using neighbor-joining analyses. Confidence in tree topology was calculated by bootstrap test amongst 1000 replicates. There were a total of 3844 positions in the final dataset.

The tree obtained from the sequences of five concatenated housekeeping gene sequences is presented in Fig. 2. Using a 100% nucleotide identity as the clustering cut-off, the ten test strains were grouped into 7 types that clustered similarly when the different genes were analyzed individually. Dissimilarities between the sequences are shown in supplementary Figure S3 along with phylogenetic trees constructed using individual genes. Three pairs of strains, MOLA1420 - MOLA1421, MOLA1578 - MOLA1596, and MOLA1611 – MOLA1612 had identical sequences for all genes sequenced in this study. Isolate MOLA1427 from *Lichina pygmaea* branched with two other isolates from the same lichen (MOLA1420 and MOLA1421) in all phylogenetic reconstructions. The five isolates from *Roccella fuciformis* always clustered together based on all 5 genes, with MOLA1615 mostly grouping with the pair MOLA1578-MOLA1596. As a general feature MOLA1488 and MOLA 1493 tended to be more distant to the remainder of the strains. Finally, no differences in topology were observed when using DNA or their translated sequences (data now shown).

### Genomic similarities

**Figure 3:**
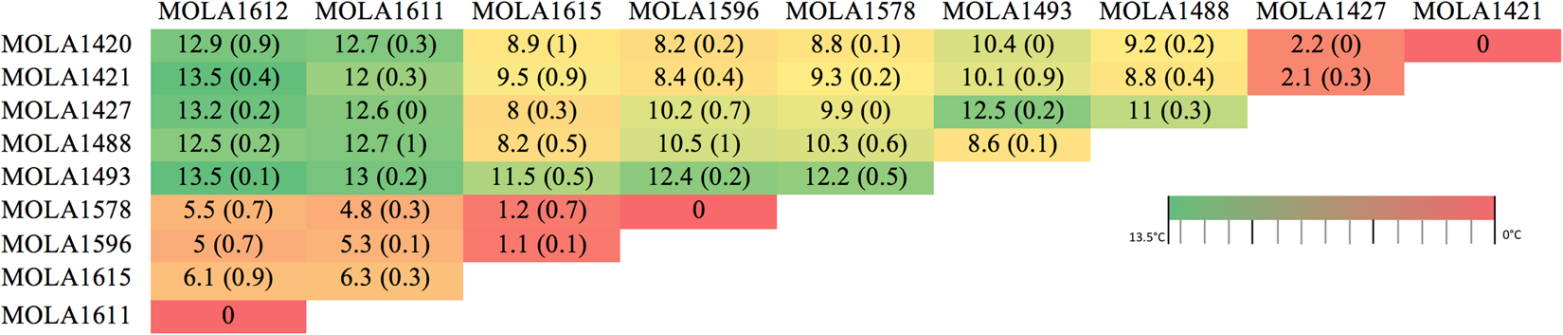
Upper triangular matrix of mean ΔTm values. ΔTm is the difference in melting temperature of the homologous genomic DNA and hybrid DNA preparation corresponding to their 50% decrease in fluorescence in melting curves. The values in the brackets are standard error of the mean between 3 replicates. Hybrid pairs showing less than 5 degree difference are considered as same genomic species.

GC content in the genomes was estimated using fluorometrically measured DNA melting curves. The GC% values of each strain are listed in Table 1. Six strains had 64% GC while the other 4 had 66%, and no systematic correlation with species, color groups or other phenotypic characteristics could be established. Based on a criteria recommended by Stackebrandt *et al*., (2002)^27^ of 5 °C difference cut-off in melting temperature between homologous and heterologous DNA-DNA hybrids the strains could be classified into 5 different genomic species (Fig. 3). The three isolates from *Lichina pygmaea* belongs to same species, whereas the two strains of *Lichina confinis* represent two additional species and finally the 5 strains from *Roccella fuciformis* represent two additional separate species.

### Metabolic profiling

Metabolic profiles were distinct among the different strains as indicated in Table S2 and Figures S4–S5. Differences between the metabolic profiles of the 10 strains are represented in Figure S4 and S5. As expected from the remainder of the analyses, MOLA1596 and MOLA 1611 were most closely related with 6 common metabolites. No obvious relationship was found between the other strains. It should be noted that MOLA1488 was again the most distinct of all, with a very high relative proportion of specific secondary metabolites not present in any other strain. High-resolution mass spectrometry allowed the putative identification of 38 natural products, while 14 metabolites were possibly novel. Among the latter, remarkably, 11 metabolites were unique to a single strain.

## Discussion

The study presented here examined the diversity between 10 isolates that were affiliated to the genus *Streptomyces* and exhibited identical SSU rRNA gene sequences. Phenotypic characteristics such as color grouping, halotolerance, pH growth range and protein coding gene sequences showed that the strains could be consistently segregated into 6 groups. Two of these groups, however, represented the same microscopic morphology and genomic species (by percentage genome similarities). This is, to best of our knowledge, the first report of bacterial strains that have 100% identical SSU rRNA sequences and that are yet diverse enough to be classified as 5 different species. Phylogenetic and metabolic diversity between *Streptomyces* strains with highly similar SSU rRNA gene sequences has been studied previously^24, 28, 29^ and such metabolic diversity within phylogenetically close strains have been also demonstrated in other actinomycetes such as *Salinispora* ^30^. The metabolic and genomic variations between strains with highly similar SSU rRNA gene sequences appear to be particularly high in the bacterial family Actinomycetaceae as noted in this study, and also in strains of family Vibrionaceae^31^. Additionally, globally widespread planktonic cyanobacteria *Synechoccocus* and *Prochlorococcus* that belong to different genera, bear completely different photosynthetic apparatus despite the high SSU rRNA similarity across the two (>98%)^32^. Nevertheless, none of these reports examined strains with identical SSU rRNA sequences, nor did they find that these bacterial strains belonged to different species.

Consequences of under-appreciating diversity between strains with identical or nearly identical SSU rRNAs genes are high. This is further exacerbated by the fact that in most studies using new generation sequencing, only a partial sequence of the gene is obtained, and sequences are clustered at operational taxonomy units at 97% sequence similarity. Here we would highlight two major repercussions that directly affect linked to human welfare. Firstly, the test organisms in this study belong to the family Actinomycetaceae, that represents the main natural source of bioactive natural products^33^. These molecules make up the largest source of pharmacological compounds (such as antibiotics) that are currently used^34^ and thus have been the main subjects of natural products drug discovery screenings. In these discovery campaigns, it is common to de-replicate the strains based on SSU rRNA gene sequences. In the likelihood that our results with *S. cyanofuscatus* is also true to other actinomycetes, de-replication based on SSU rRNA sequences would result in overlooking possible novel chemical structures with drug potential, as it was the case for 14 metabolites from 6 analyzed metabolomes. In the case of *Vibrionaceae*, the SSU sequences of several species that are pathogenic to humans and to marine invertebrates of commercial importance, are in some case nearly identical to those of non-pathogenic species (i.e. *V. parahaemolyticus* and *V. natriegens* ^35^), which severely hampers the use of SSU rRNA and its coding gene for environmental monitoring.

Finally, it was very interesting to note that many of the phenotypical characteristics of the strains, protein coding sequences and the secondary metabolic patterns were most similar between strains isolated from the same lichen species. This is in line with earlier studies that have shown that phenotypical (and by extension and most likely genomic) similarity might be higher in sympatry^24, 36^, and they could indicate niche-specific adaptation^24, 37, 38^. Alternatively (or in addition) this phenotypical and genomic similarity between sympatric strains might be due to an increased opportunity for horizontal gene exchange, a phenomenon that is well known to occur in *Streptomyces* ^39^.

## Conclusion

A polyphasic approach was used to assess phenotypic, morphology and genomic relationship between 10 strains with identical SSU rRNA gene sequences. We demonstrate that despite this identity, the strains group as 5 different species. The results hold significance to the increased use of SSU rRNA gene sequences as proxies of prokaryotic diversity. We also show that the biosynthetic properties varied between the 5 species.

## Methods

### Source of isolates

A total of ten bacterial strains with identical SSU rRNA gene sequence were used in this study. The strains were isolated from 3 different coastal lichens; *Lichina pygmaea, Lichina confinis* and *Roccella fuciformis* (Table 1). Details of isolation protocols are described by Parrot and collaborators^25^. Briefly, the lichen samples were aseptically crushed, washed with sterile seawater and ground to form a homogenate. The wash water and the homogenate were used as different inoculant on actinomycetes isolation agar (AIA, Difco^TM^, France) and marine agar (MA, Difco^TM^, France), both media supplemented with nalidixic acid (Sigma-Aldrich, France) and cycloheximide (Sigma-Aldrich, France). The isolates were deposited in the MOLA culture collection (WDCM911, Collection of Microbial Observatory Laboratoire Arago, France). All 10 isolates of interest have identical SSU rRNA gene sequences to that of *Streptomyces cyaneofuscatus* type strain JCM 4364 ^T^.

### Preparation of spore suspension

The strains were cultured on International Streptomyces Project medium 3 (ISP 3; oat meal agar^40, 41^) for 21 to 30 days. The spores were collected by scrapping over top of the colonies and suspended in sterile phosphate buffered saline solution. The preparation was passed through sterile cotton wool in a syringe to remove the hyphal matrix. Numbers of viable spores were estimated number of colonies formed when plated on nutrient agar media. The spore suspensions were maintained at −20 °C for not more than 2 weeks. Before use the suspensions were thawed at 4 °C.

### Halotolerance and pH growth range

Halotolerance of the isolates were estimated by plotting growth curves at differing pH and salt concentrations. International Streptomyces Project media 2 (ISP 2) broths were prepared with three NaCl concentrations (5%, 10% & 20%) and three different pH values (5.0, 7.0 & 9.0). The pH tests were carried out in broths that had 10% NaCl and the halotolerance tests were done at in media at pH 7.0.

Spore suspensions (~10^2^ viable spores) of the strain were inoculated into 9 tubes of each media and incubated in an orbital shaker at 28 °C at 120 rpm. For each strain, 3 tubes of every media were removed for analyses after 7, 14 and 21 days. Each tube represented a replicate (among triplicates) for each strain per condition. The broth was individually filtered through pre-weighed Whatman^®^ cellulose filter paper (Grade 1). The harvested biomass oven dried at 55 °C for 24 h. Total dry weights of each of the triplicates per condition for each strain were recorded and plotted against time to evaluate tolerance to pH and salt.

### Color grouping

The test strains were grouped according to their pigment production characteristics as recommended by Williams *et al*., 1969. A computer-assisted numerical method recommended by Antony-Babu and colleagues^42^ was used since this method could be more objective than the protocol first proposed in 1969^23^. The 10 test isolates were inoculated as spore suspension (~50 spores) onto ISP 3 and International *Streptomyces* Project media 6 (ISP 6, Difco, France^43^) as triplicate streak cultures. ISP 3 plates were incubated 21 days and ISP 6 for 4 days, both at 28 °C. At the end of 4 days ISP 6 media cultures were checked for melanin pigment production. The ISP 3 plates were examined by eye for aerial spore mass color, substrate mycelium pigmentation and the color of any soluble pigments after 7, 14 and 21 days. The isolates were grouped based on similarities in the colony color properties.

### Morphological examination

Spore chain arrangements of the strains were examined microscopically. To encourage spore formation, the strains were inoculated (~50 spores) onto ISP 3 media. Sterile coverslips were inserted into the agar surface in a 45° angle, with the bottom face leaning over the inoculum. The set-up was incubated at 28 °C for 21 days. After the incubation period, the coverslips were carefully removed and stained with methyl violet stain. The spore chain morphology was examined under 100 X magnification using a light microscope.

### Genomic DNA isolation

In order to extract genomic DNA, the test strains were cultured on media described by Antony-Babu and Goodfellow^26^. This media allows growth of streptomycetes on agar surfaces that are easily detached from the substrate mycelium. The aerial biomass was harvested after 5 days using sterile toothpicks without touching the agar. The cells were first briefly rinsed with 5 mM EDTA (pH 8.0) and then with 99% ethanol. The pelleted biomass was resuspended in 10% SDS and mixed thoroughly. The suspension was heat-lysed at 80 °C for 20 minutes. The lysate was extracted using a conventional phenol: chloroform: isoamylalcohol protocol, followed by chloroform: isoamyl alcohol extraction and ethanol precipitation. The dried pellets were suspended in molecular grade DNase and RNase free water. The genomic DNA extracts were checked for quality & quantity on agarose gel and NanoVue spectrophotometer (GE Healthcare) and stored at −20 °C until further use.

### Protein coding gene sequencing

Genomic DNA samples were used as templates for PCR amplification of the protein-coding housekeeping genes: *atpD*, *gyrB, recA*, *rpoB* and *trpB* using PCR primers previously described by Guo and colleagues^21^. The original conditions described by the authors were modified to accommodate the use of a fast PCR reaction mix KAPA2G (Clinisciences, Nanterre France), where the denaturation, annealing and amplification steps were carried out for 15, 15 and 30 seconds respectively. Amplicons were purified using Agencourt^®^ AMPure^®^ XP Kit (Beckman Coulter, Villepinte, France) and used in sequencing reactions were carried out with Big Dye Terminator (V3.1; Life Technologies) using internal sequencing primers^21^ as well as the PCR primers used to synthetize the amplicons. Cycle sequencing products were cleaned using Agencourt^®^ CleanSeq^®^ Kit (Beckman Coulter) and sequenced using ABI 3130xl genetic Analyser Sequencer (Life Technologies). Partial sequences were assembled into contigs using MEGA 5.1 and for each of the housekeeping gene, the sequences available on the Genbank database that were less than 2000 bp and attributed to genus *Streptomyces* were retrieved. Contigs were aligned along with the database sequences using CLUSTAL X^44^. Neighbor-joining phylogenetic trees were built using distance matrices obtained using the Kimura-2 parameter model and their robustness evaluated from 1000 trees built using bootstrap resampling.

### Genomic GC%

G + C content of the strains were obtained fluorimetrically based on the procedure described by De Ley and Tijtgat (1970)^45^. The original method was downscaled to 20 µl with 0.5 µg of each genomic DNA suspended in a 0.1 ml optical clear tubes along with 0.1x SSC buffer (pH 8) and 1x SYBR Gold. A thermal ramp of 25 °C to 100 °C with a 1 °C rise per minute was performed using a StepOnePlus™ Real-Time PCR system (Applied Biosystems). The fluorescence at 300 nm was measured at each step of the thermal ramp and the *T*_m_ of each strain calculated from the minimum value of the slope of the tangent to the melting curve of fluorescence versus temperature. The %G + C of each strain was calculated using the equation: %G + C = (1.99*T*_m_) − 71.08.

### Genomic similarities

The optimum temperature of renaturation (*T*_or_) was calculated using the GC% obtained earlier, by applying the formula *T*_or_ = 0.51(%G + C) + 47. As with the GC content protocol, the reaction volumes were downscaled to 20 µl. Genomic DNA hybrids were generated by mixing 0.5 µg each of genomic DNA from the reference and the test strain and suspended with 0.1x SSC buffer (pH 8) in a 0.1 ml optical clear tubes (for a total volume of 18 µl). The homologous DNA control and hybrid DNA samples were denatured and re-annealed using in a StepOnePlus™ Real-Time PCR system (Applied Biosystems) using the following conditions: 99 °C for 10 minutes, *T*_or_ for 8 hours followed by progressive steps in which the temperature was dropped by 10 °C then held for 1 hour until 25 °C was achieved. 2 µl SYBR Gold was added to this reaction mix to achieve a final 0.1 X concentration of the dye and 1 X concentration of SSC in the final mix. The thermal denaturation was achieved as follows: 25 °C for 15 minutes, followed by a thermal ramp from 25–100 °C with a 1 °C rise per minute, fluorescence measurements were taken at each step of the ramp. The *T*_m_ of the homologous and hybrid DNA preparations were calculated by measuring the temperature corresponding to a 50% decrease in fluorescence in the melting curve of fluorescence versus temperature. Δ*T*_m_ is the difference between the temperatures of homologous and heterologous genomic DNA mixes; differences of 5 °C or more are considered to show that the tested strains belong to different genomic species^46, 47^.

### Metabolic profiling

#### Extraction of metabolites

Representative strains from the 6 color groups (MOLA1420, MOLA1427, MOLA1488, MOLA1493, MOLA1596 & MOLA1611) were used in this analysis. Inocula (50 µl) from PBS spore suspensions were spread over trypic soy agar plates and incubated at 28 °C for 15 days. Entire plate contents were shredded into small pieces (~0.5 cm^3^) using a sterile scalpel. The agar blocks were suspended in 15 ml of HPLC grade ethyl acetate (Carlo Erba, France). The preparation was shaken overnight in the dark at 5 °C on an orbital shaker. The liquid phase was removed, dehydrated with addition of sodium sulfate (~1.0 g) and filtered through cotton wool. The extracts were evaporated in a HT4X rotatory vacuum concentrator (Genevac, Ipswich, UK) system over night at 5 °C. Ethyl acetate extracts were treated prior to analysis using solid phase extraction (Waters Sep-Pak C18, Vac 1 cm^3^, 100 mg). The stationary SPE phase was activated with 2 mL of acetonitrile. The cartridge was loaded with <5.0 mg of each extract, which was solubilized in 500 µL of acetonitrile. The elution was performed with 1 mL acetonitrile to eliminate the most lipophilic metabolites. The eluates were dried in a Genevac system and were dissolved in acetonitrile to afford yield 1.5 mg/mL solutions for profiling analyses.

#### Profiling

The composition of the extracts was analyzed with a Waters Acquity Ultra High Pressure Liquid Chromatography system coupled to a Waters Micromass LCT Premier Time-of-Flight mass spectrometer (Milford, MA, USA) equipped with an electrospray interface. The UHPLC system was equipped with a Waters Acquity UPLC BEH C18 column (150 mm × 2.1 mm, 1.7 µm). The oven temperature was maintained at 40 °C and the solvent flow was 0.6 mL/min. The mobile phases were H_2_O (A) and acetonitrile (B), each containing 0.1% formic acid (v/v), with the following gradient: 90:10 (0–1 min), 10 to 100% B (1–20 min), and 100% B for 10 min. Analysis of each extract (1.0 µL injected) was performed in both positive (ESI^+^) and negative ionization (ESI^–^) modes in the 80–1500 Da range. The other ESI conditions were set as follows: capillary voltage 2800 V, cone voltage 40 V, source temperature 120 °C, desolvation temperature 330 °C, cone gas flow 20 L/h, desolvation gas flow 600 L/h, and MCP (micro-channel plate) detector voltage 2400 V. Profiles with ESI+ were best for automated analysis.

The UHPLC-MS chromatograms were converted into the cdf format using the Databridge^TM^ application provided with MassLynx^TM^ (Waters, Guyancourt, France). Peak selection and peak alignment were performed with MzMine 2.10^48^. The extract from cultured medium was included in the analysis to exclude its peaks from the profiles. The removal of peaks was performed manually, and in some cases, peaks that were too low to be detected were added manually if present because the objective of the profiling analysis was to detect biosynthetic pathways no matter their level of expression. Subsequently aligned and cleaned data was exported to Microsoft® Excel (2007) yielding a 6 (N) × 52 (X) data matrix. Peak height information was removed and replaced by 1 if compound was present and 0 if absent. Chemometric data analyses were done using Addinsoft® XLSTAT version 2013.4.08. Hierarchical clustering analysis (HCA) was performed to evaluate the Euclidean distance between all profiles.

Putative identification of the metabolites produced from six *Streptomyces cyaneofuscatus* strains was performed. Presumptive molecular formula and the difference between the exact mass and the experimental data (Δ ppm) were determined using MassLynx^TM^. The identification of compounds was proposed using three databases (Antibase2012, Dictionary of Natural Products and Reaxys) focusing on natural compounds already isolated from microorganisms, fungi, sponges and some few algae based on the origin of these six bacterial strains.

### Data availability statement

The datasets generated during and/or analysed during the current study are available from the corresponding author on reasonable request.

## Electronic supplementary material

Supplementary information
