# Supplementary material for: Multiple Streptomyces species with distinct secondary metabolomes have identical 16S rRNA gene sequences
Source: Sci Rep. 2017 Sep 11;7:11089. doi: 10.1038/s41598-017-11363-1 (PMC5593946; doi:10.1038/s41598-017-11363-1)
Supplement: Supplementary file 1 — Supplementary information [file 41598_2017_11363_MOESM1_ESM.pdf]

Supplementary Information

Multiple *Streptomyces* species with distinct secondary metabolomes have identical 16S rRNA gene sequences

Sanjay Antony-Babu[1,4], Didier Stien[1], Véronique Eparvier[2] and Delphine Parrot[3], Sophie Tomasi[3] and Marcelino T Suzuki[1]*

1. *Sorbonne Universités, UPMC Univ Paris 06, CNRS, Laboratoire de Biodiversité et Biotechnologies Microbiennes (LBBM), Observatoire Océanologique, F-66650 Banyuls/Mer, France CNRS, USR 3579, LBBM, Observatoire Océanologique, 66650 Banyuls-sur-Mer, France*
2. *CNRS, Institut de Chimie des Substances Naturelles, 91198 Gif-sur-Yvette cedex, France*
3. *UMR CNRS 6226, Institut des Sciences Chimiques de Rennes, Equipe PNSCM "Produits naturels – Synthèses – Chimie Médicinale", UFR Sciences Pharmaceutiques et Biologiques, Univ. Rennes 1, Université Bretagne Loire, 2 Avenue du Pr. Léon Bernard, F-35043 Rennes, France*
4. *Current address and affiliation: Department of Food Sciences and Technology, University of Nebraska-Lincoln, Lincoln, Nebraska, 68504, United States of America*

[*] Corresponding author. Tel.: +33 (0) 430192401

E-mail address: suzuki@obs-banyuls.fr (M.T. Suzuki)

Figure S1: Growth curves of the ten tested strains in different conditions. X axis denotes the time of sampling in days (7, 14 and 21 days) and Y axis denotes dry weight biomass in grams.

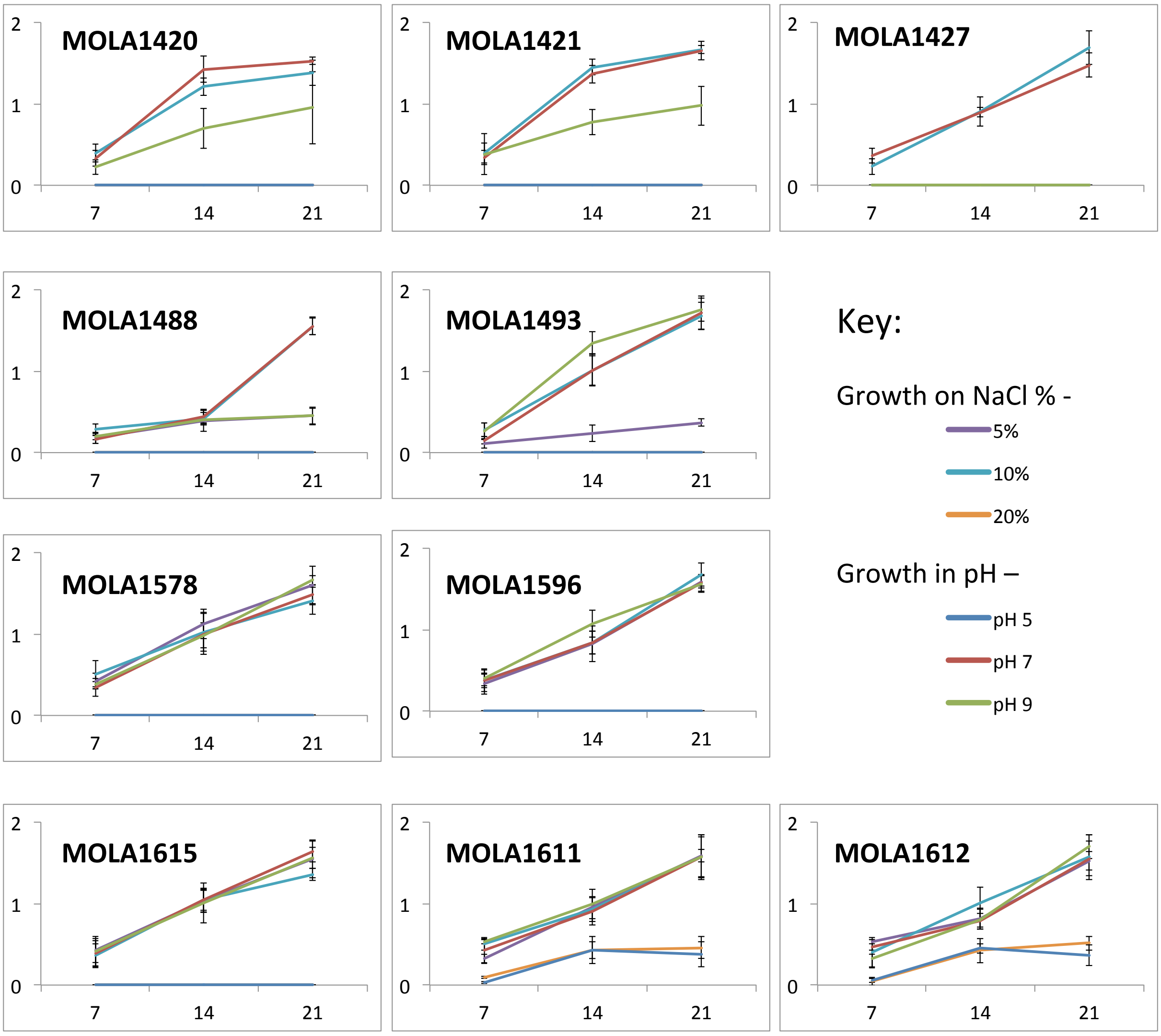

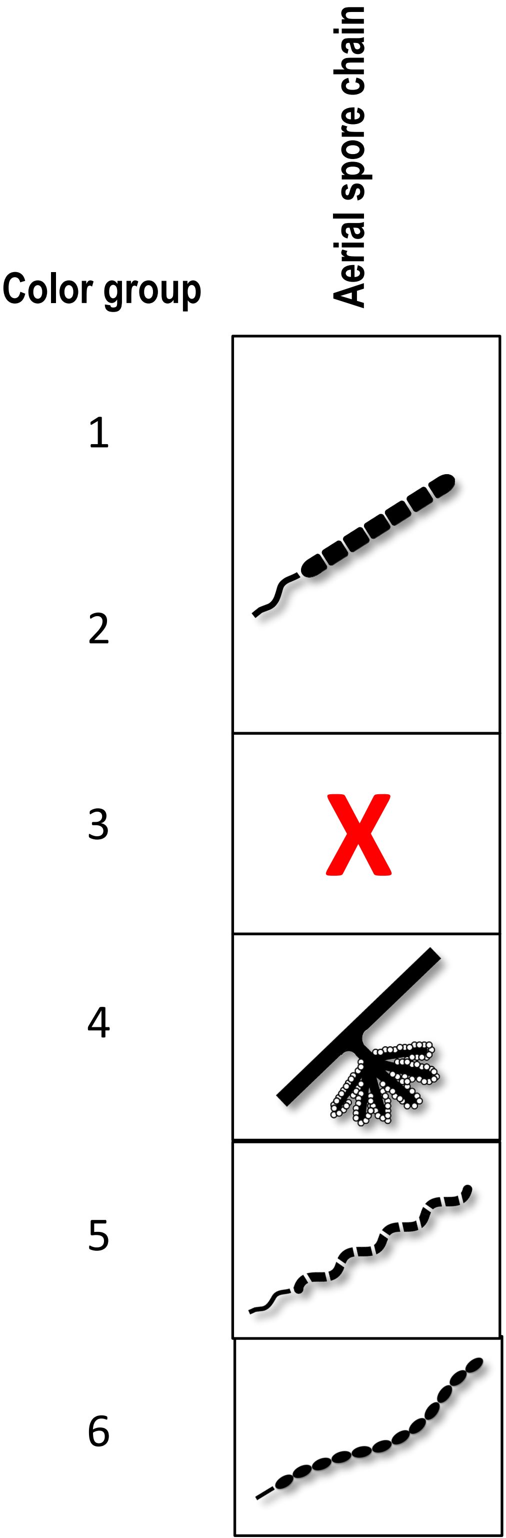

Figure S2: Micromophology of spore chain arrangement has been correlated with the color groups.

Kimura-2 parameter distance | **Percentage nucleotide differences**

atpD

Kimura-2 parameter distance

	MOLA1612	MOLA1611	MOLA1615	MOLA1596	MOLA1578	MOLA1493	MOLA1488	MOLA1427	MOLA1421
MOLA1420	0.0104	0.0104	0.0091	0.0091	0.0091	0.0065	0.0143	0.0130	0.0000
MOLA1421	0.0104	0.0104	0.0091	0.0091	0.0091	0.0065	0.0143	0.0130	
MOLA1427	0.0130	0.0130	0.0117	0.0117	0.0117	0.0143	0.0196		
MOLA1488	0.0143	0.0143	0.0130	0.0130	0.0130	0.0104			
MOLA1493	0.0065	0.0065	0.0052	0.0052	0.0052				
MOLA1578	0.0039	0.0039	0.0026	0.0000					
MOLA1596	0.0039	0.0039	0.0026						
MOLA1615	0.0039	0.0039							
MOLA1611	0.0000								

Percentage nucleotide differences

	MOLA1612	MOLA1611	MOLA1615	MOLA1596	MOLA1578	MOLA1493	MOLA1488	MOLA1427	MOLA1421
MOLA1420	1.03	1.03	0.90	0.90	0.90	0.65	1.42	1.29	0.00
MOLA1421	1.03	1.03	0.90	0.90	0.90	0.65	1.42	1.29	
MOLA1427	1.29	1.29	1.16	1.16	1.16	1.42	1.94		
MOLA1488	1.42	1.42	1.29	1.29	1.29	1.03			
MOLA1493	0.65	0.65	0.52	0.52	0.52				
MOLA1578	0.39	0.39	0.26	0.00					
MOLA1596	0.39	0.39	0.26						
MOLA1615	0.39	0.39							
MOLA1611	0.00								

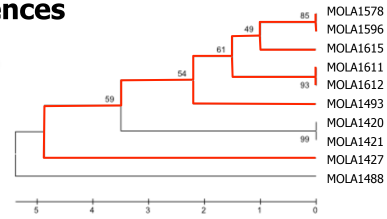

gyrB

Kimura-2 parameter distance

	MOLA1612	MOLA1611	MOLA1615	MOLA1596	MOLA1578	MOLA1493	MOLA1488	MOLA1427	MOLA1421
MOLA1420	0.0539	0.0539	0.0579	0.0513	0.0513	0.0539	0.0316	0.0252	0.0000
MOLA1421	0.0539	0.0539	0.0579	0.0513	0.0513	0.0539	0.0316	0.0252	
MOLA1427	0.0633	0.0633	0.0687	0.0674	0.0674	0.0647	0.0473		
MOLA1488	0.0513	0.0513	0.0593	0.0526	0.0526	0.0540			
MOLA1493	0.0227	0.0227	0.0304	0.0239	0.0239				
MOLA1578	0.0163	0.0163	0.0062	0.0000					
MOLA1596	0.0163	0.0163	0.0062						
MOLA1615	0.0201	0.0201							
MOLA1611	0.0000								

Percentage nucleotide differences

	MOLA1612	MOLA1611	MOLA1615	MOLA1596	MOLA1578	MOLA1493	MOLA1488	MOLA1427	MOLA1421
MOLA1420	0.99	0.99	0.87	0.87	0.87	0.62	1.36	1.24	0.00
MOLA1421	0.99	0.99	0.87	0.87	0.87	0.62	1.36	1.24	
MOLA1427	1.24	1.24	1.12	1.12	1.12	1.36	1.86		
MOLA1488	1.36	1.36	1.24	1.24	1.24	0.99			
MOLA1493	0.62	0.62	0.50	0.50	0.50				
MOLA1578	0.37	0.37	0.25	0.00					
MOLA1596	0.37	0.37	0.25						
MOLA1615	0.37	0.37							
MOLA1611	0.00								

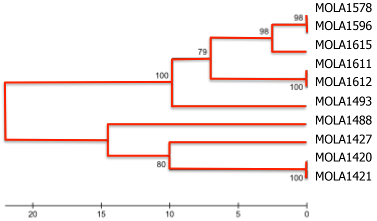

recA

Kimura-2 parameter distance

	MOLA1612	MOLA1611	MOLA1615	MOLA1596	MOLA1578	MOLA1493	MOLA1488	MOLA1427	MOLA1421
MOLA1420	0.0502	0.0502	0.0472	0.0487	0.0487	0.0579	0.0441	0.0291	0.0000
MOLA1421	0.0502	0.0502	0.0472	0.0487	0.0487	0.0579	0.0441	0.0291	
MOLA1427	0.0396	0.0396	0.0381	0.0381	0.0381	0.0518	0.0457		
MOLA1488	0.0352	0.0352	0.0412	0.0367	0.0367	0.0626			
MOLA1493	0.0595	0.0595	0.0610	0.0579	0.0579				
MOLA1578	0.0014	0.0014	0.0115	0.0000					
MOLA1596	0.0014	0.0014	0.0115						
MOLA1615	0.0101	0.0101							
MOLA1611	0.0000								

Percentage nucleotide differences

	MOLA1612	MOLA1611	MOLA1615	MOLA1596	MOLA1578	MOLA1493	MOLA1488	MOLA1427	MOLA1421
MOLA1420	1.14	1.14	1.00	1.00	1.00	0.71	1.57	1.43	0.00
MOLA1421	1.14	1.14	1.00	1.00	1.00	0.71	1.57	1.43	
MOLA1427	1.43	1.43	1.29	1.29	1.29	1.57	2.14		
MOLA1488	1.57	1.57	1.43	1.43	1.43	1.14			
MOLA1493	0.71	0.71	0.57	0.57	0.57				
MOLA1578	0.43	0.43	0.29	0.00					
MOLA1596	0.43	0.43	0.29						
MOLA1615	0.43	0.43							
MOLA1611	0.00								

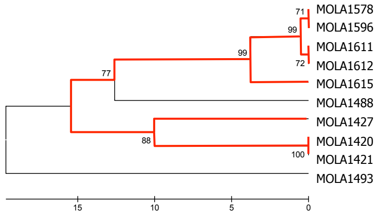

rpoB

Kimura-2 parameter distance

	MOLA1612	MOLA1611	MOLA1615	MOLA1596	MOLA1578	MOLA1493	MOLA1488	MOLA1427	MOLA1421
MOLA1420	0.0322	0.0322	0.0309	0.0322	0.0322	0.0370	0.0273	0.0165	0.0000
MOLA1421	0.0322	0.0322	0.0309	0.0322	0.0322	0.0370	0.0273	0.0165	
MOLA1427	0.0285	0.0285	0.0273	0.0285	0.0285	0.0309	0.0201		
MOLA1488	0.0225	0.0225	0.0225	0.0225	0.0225	0.0297			
MOLA1493	0.0309	0.0309	0.0297	0.0309	0.0309				
MOLA1578	0.0000	0.0000	0.0023	0.0000					
MOLA1596	0.0000	0.0000	0.0023						
MOLA1615	0.0023	0.0023							
MOLA1611	0.0000								

Percentage nucleotide differences

	MOLA1612	MOLA1611	MOLA1615	MOLA1596	MOLA1578	MOLA1493	MOLA1488	MOLA1427	MOLA1421
MOLA1420	0.93	0.93	0.82	0.82	0.82	0.58	1.28	1.17	0.00
MOLA1421	0.93	0.93	0.82	0.82	0.82	0.58	1.28	1.17	
MOLA1427	1.17	1.17	1.05	1.05	1.05	1.28	1.75		
MOLA1488	1.28	1.28	1.17	1.17	1.17	0.93			
MOLA1493	0.58	0.58	0.47	0.47	0.47				
MOLA1578	0.35	0.35	0.23	0.00					
MOLA1596	0.35	0.35	0.23						
MOLA1615	0.35	0.35							
MOLA1611	0.00								

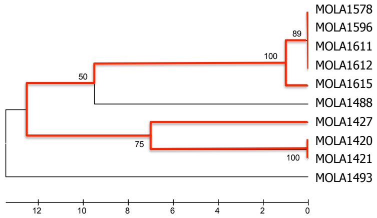

trpD

Kimura-2 parameter distance

	MOLA1612	MOLA1611	MOLA1615	MOLA1596	MOLA1578	MOLA1493	MOLA1488	MOLA1427	MOLA1421
MOLA1420	0.0556	0.0556	0.0587	0.0556	0.0556	0.0587	0.0406	0.0361	0.0000
MOLA1421	0.0556	0.0556	0.0587	0.0556	0.0556	0.0587	0.0406	0.0361	
MOLA1427	0.0511	0.0511	0.0526	0.0511	0.0511	0.0617	0.0466		
MOLA1488	0.0421	0.0421	0.0436	0.0421	0.0421	0.0421			
MOLA1493	0.0346	0.0346	0.0346	0.0346	0.0346				
MOLA1578	0.0000	0.0000	0.0042	0.0000					
MOLA1596	0.0000	0.0000	0.0042						
MOLA1615	0.0042	0.0042							
MOLA1611	0.0000								

Percentage nucleotide differences

	MOLA1612	MOLA1611	MOLA1615	MOLA1596	MOLA1578	MOLA1493	MOLA1488	MOLA1427	MOLA1421
MOLA1420	0.99	0.99	0.87	0.87	0.87	0.62	1.36	1.24	0.00
MOLA1421	0.99	0.99	0.87	0.87	0.87	0.62	1.36	1.24	
MOLA1427	1.24	1.24	1.12	1.12	1.12	1.36	1.86		
MOLA1488	1.36	1.36	1.24	1.24	1.24	0.99			
MOLA1493	0.62	0.62	0.50	0.50	0.50				
MOLA1578	0.37	0.37	0.25	0.00					
MOLA1596	0.37	0.37	0.25						
MOLA1615	0.37	0.37							
MOLA1611	0.00								

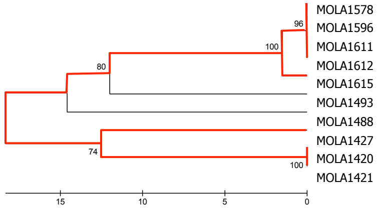

Figure S3. Properties of each of protein coding loci examined. The first two columns contain upper triangular matrices of dissimilarities based on Kimura-2 parameter and percentage of number of nucleotides. The last column shows UPGMA tree based on 1000 replicates (bootstrap). The branching patterns that were in agreement with those that were obtained by maximum parsimony, neighbor joining, maximum likelihood and minimum evolution are shown in red.

Figure S4. Aligned metabolomic profiles of actinomycete culture extracts. The x-axis indicate the retention time. Peak intensities were all set to 1. Culture medium compound peaks have been removed from the profiles so that only microbial metabolites remain.

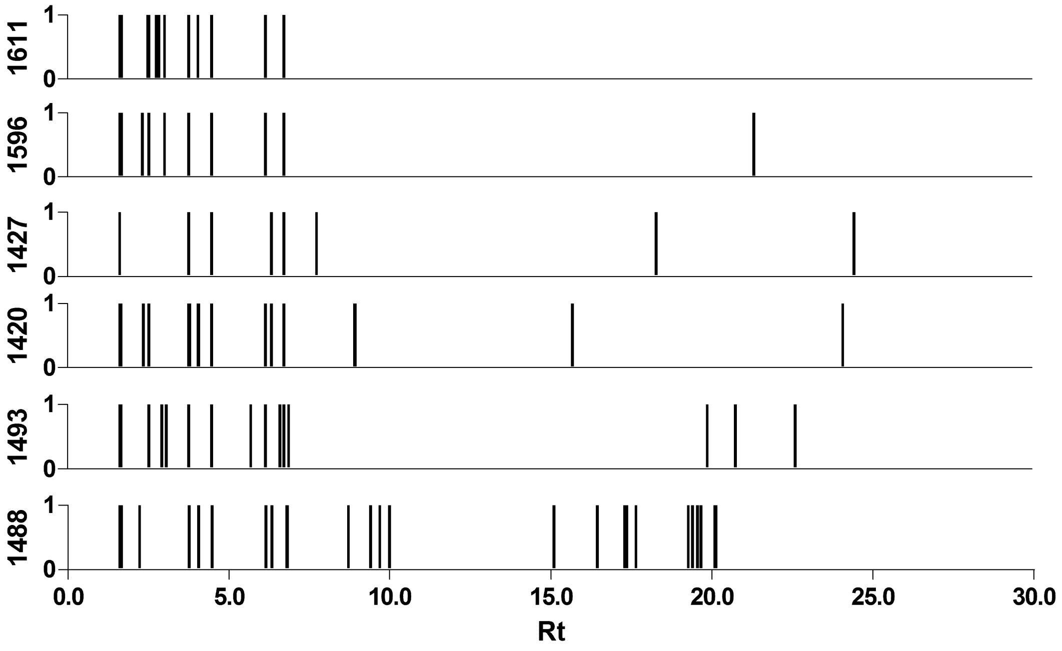

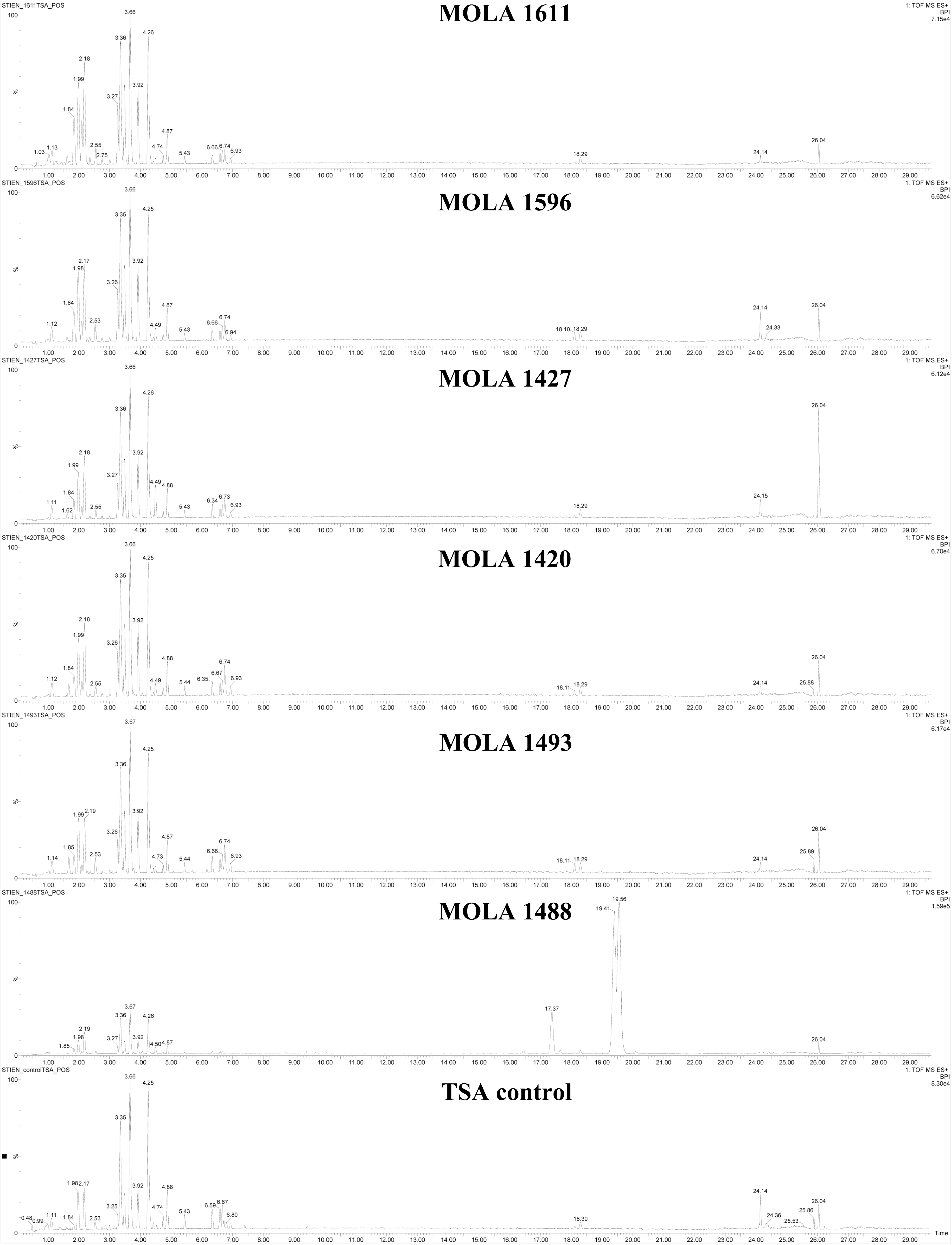

Figure S5 Aligned raw UHPLC-HRMS total ion current profiles of culture extracts of selected *S. cyaneofuscatus* strains grown in TSA. TSA medium extract profile is shown for comparison. The x-axis indicates the retention time and the y-axis the percent of peak area relative to the highest peak

Table S1: Color group characters of the 10 test strains after 21 days incubation. The numerical values denote the average red, green and blue values of colors of aerial spore mass, diffusible pigment and colony reverse on ISP 3. The colors of the average RGB values are used as fill-colors for the respective cells in the table. Melanin pigment production was concluded based on production of dark pigment on ISP 6 media.

**Color group number**	**Characteristics on ISP 3 (average *red:green:blue* color values)**			**Melanin pigment production on ISP 6**	**Strains**
	Aerial spore mass color	Color of diffusible pigment	Colony reverse color		
1	180:219:196	138:127:75	228:209:168	+	MOLA 1420
	195:209:205	150:134:80	215:207:165	+	MOLA 1421
2	154:184:164	112:95:46	219:184:127	+	MOLA 1427
3	201:202:165	173:129:4	241:188:46	+	MOLA 1488
4	192:209:201	88:96:89	175:158:135	+	MOLA 1493
5	165:181:155	84:101:83	178:194:169	-	MOLA 1578
	153:157:125	86:102:88	181:197:173	-	MOLA 1596
	181:192:156	85:104:91	179:196:172	-	MOLA 1615
6	111:111:77	95:112:101	159:185:173	-	MOLA 1611
	121:118:79	93:114:99	160:184:180	-	MOLA 1612

Table S2: Putative identification, sources and presence (+)/absence (-) for compounds highlighted in six *Streptomyces cyaneofuscatus* strains.

[1]Δ ppm stands for the relative m/z difference between the experimental and the calculated m/z for the $[M+H]^+$ or the $[M+Na]^+$ ion, as stated in the ion type column.
[3]The high resolution mass of this compound was incorrect due to superimposition with other signals. *N*-Methyl-dactinomycin was isolated and unambiguously identified by NMR.

Rt (min)	*m/z*	Ion type	Calcd. m/z ($[M+H]^+$ or	Putative Molecular Formula (M)	Δ ppm[1]	Putative Identification[2] and Sources	MOLA 1427	MOLA 1493	MOLA 1420	MOLA 1596	MOLA 1488	MOLA 1611	References
1.63	**146.0605**	**$[M+H]^+$**	146.0606	C_9H_7NO	0.5	*/***Indole-3-carbaldehyde (*Streptomyces zhaozhouensis*; *Aspergillus versicolor*; *Penicillium* sp.)	+	+	+	+	+	+	1-4
1.68	**212.1401**	**$[M+H]^+$**	212.1399	$C_{10}H_{17}N_3O_2$	1.0	***Bacillusamide A (*Anthocidaris crassispina* derived *Bacillus* sp.)	-	-	-	+	-	+	5
1.68	**152.0711**	**$[M+H]^+$**	152.0712	$C_8H_9NO_2$	0.4	**p*-Hydroxyphenyl-acetamide (marine *Cytophaga* sp.; marine *Vibrio parahaemolyticus*); *Anthranilic acid methyl ester (*Streptomyces avermitilis*); */***4-Hydroxyacetanilide (*Streptomyces venezuelae*); ***2-(Acetylamino)phenol (*Streptomyces* sp.; *Penicillium* sp.; *Actinomyces* sp.); ****N*-Methylanthranilic acid (*Streptomyces* sp.); ****N*-Methyl-*p*-aminobenzoic acid (*Streptomyces* sp.); ***2-(4-Hydroxyphenyl)acetamide (*Cytophaga* sp.); **/***(2-Amino-phenyl)-acetic acid (*Colletotrichum gloeosporioides*); **/***4-Hydroxyphenylacetaldehyde oxime (*Streptomyces nigellus*); */***Streptokordin (*Streptomyces* sp.)	-	+	+	-	+	-	6-18
2.24	**136.0761**	**$[M+H]^+$**	136.0762	C_8H_9NO	0.9	*/****p*-(Methylamino)-benzaldehyde (*Camarophyllus virgineus*); *2-Phenylacetamide (*Streptomyces alboniger, sponges, cyanobacterium*); *1-(2-Amino-phenyl)-ethanone (marine *Streptomyces, Saccharomyces cerevisiae*); *3-Aminophenyl 1-ethanone (*Phlebia radiata*); ***Acetanilid (*Streptosporangium* sp.); ***Benzeneacetamide (*Strep tomyces* sp.; *Streptoverticillium olivoreticuli; Vibrio parahaemolyticus*); ***Agrocybyne A (*Agrocybe praecox*)	-	-	-	-	+	-	6, 19-30
2.33	**160.0767**	**$[M+H]^+$**	160.0762	$C_{10}H_9NO$	2.6	**/***4-Hydroxy-2-methylquinoline (*Pseudomonas aeruginosa*) ; */**/*** 4-(Hydroxymethyl)quinoline (*myxobacteria*), **/*** (*E*) or (*Z*)-3-Ethylidene-1,3-dihydroindol-2-one (*Colletotrichum fragariae* Brooks), *Quinoline-2-methanol (*Kitasatoa griseophaeus*); *8-Amino-2-naphthalenol (cyanobacterium *Aphanizomenon flos-aquae*)	-	-	-	+	-	-	20, 31-33
2.37	**185.1291**	**$[M+H]^+$**	185.1290	$C_9H_{16}N_2O_2$	0.6	*/**/***Cyclo (L-Ala-L-Leu) (various microorganisms) (e.g. *Streptomyces* sp. YIM 56130)	-	-	+	-	-	-	34
2.50	**138.0553**	**$[M+H]^+$**	138.0555	$C_7H_7NO_2$	1.5	*/**/***Pyrrole-3-yl-2-propenoic acid (*Streptomyces parvulus Tue 2480)*; */**/*** *N*-Formyl-2-aminophenol (Penicillium *notatum-Westling)* ; */**/****p*-Aminobenzoic acid (Hansenula *anomala, Mycotoru* la *lipolytica, marine S* treptomyc*es sp. B8459*), **/****o*-Aminobenzoic acid (various bacteria e.g. *Streptomyces* sp. IFM 11299, S. sp GW63/*1* 571 ; limnic strain GW73A, *S. staurosporeus* + tryptophan, *Paenibacillus polymyxa* +/- tryptophan); *Anthranilic acid (widespread in bacteria) ; */****p*-Hydroxybenzamide *(Streptomyces tendae);* */**/***Trigonelline and Homarine (sponges, *Polyporus sulfureus*)	-	-	-	-	-	+	19, 35-41
2.53	**229.1018**	**$[M+H]^+$**	229.1011	$C_{10}H_{16}N_2O_2S$	3.3	*/**Cyclo(L-Pro-L-Met) (sponge-associated *Pseudomonas aeruginosa*)	-	+	+	+	-	+	42
2.76	**219.1139**	**$[M+H]^+$**	219.1134	$C_{12}H_{14}N_2O_2$	2.4	*/**/***PD 125375 (*Streptomyces* sp); */***Cyclo(L-Ala-L-Phe) (various bacteria); ***(2*S*,3*R*)-threo-β-Methyl-L-tryptophan, erythro-β-Methyl-L-tryptophan*** (*Streptomyces flocculus*), β-Hydroxy-*N*-β-acetyltryptamine (*Streptomyces staurosporeus*); 5-Hydroxy-*N*-β-acetyltryptamine (*Streptomyces staurosporeus*); */*** (3a*R*,8a*S*)-1-Acetyl-1,2,3,3a,8,8a-*H*-hexahydropyrrolo[2,3-b]indol-3a-ol (*S. staurosporeus* + tryptamine hydrochloride); *(3a*R*,8a*R*)-3a-Acetoxy-1,2,3,3a,8,8a-hexahydropyrrolo[2,3-b]indol (endophytic *Fusarium incarnatum* HKI00504 from mangrove) ; ***Damirone A (*Zyzzia fuliginosa*)	-	-	-	-	-	+	43-47
2.85	**128.1075**	**$[M+H]^+$**	128.1075	$C_7H_{13}NO$	0.5	no hit from microorganisms	-	-	-	-	-	+	

2.94	**118.0326**	**Fragment of $[M+H]^+$ at *m/z* 207.0636**	207.0626	$C_7H_{14}N_2OS_2$	4.9	no hit in antibase/DNP/Reaxys	-	+	-	-	-	-	
3.02	**300.1355**	**$[M+H]^+$**	300.1348	$C_{16}H_{17}N_3O_3$	2.1	*/**/***3β-Hydroxy-cyclo-L-tryptophyl-L-proline (*Aspergillus fumigatus*), **/***Cyclo(4-hydroxy-*S*-Pro-*S*-Trp) (marine-derived *Streptomyces* sp)	-	-	-	+	-	+	48, 49
3.08	**177.0671**	**n.d.**	n.d.	n.d.	n.d.	n.d.	-	+	-	-	-	-	
3.78	**217.0983**	**$[M+H]^+$**	217.0977	$C_{12}H_{12}N_2O_2$	2.5	*/**/****N*-Acetyl-β-oxotryptamine (Marine *Janibacter limosus*; *Streptosporangium* sp.; *Bacillus pumilus*); */**/***Damirone A (Sponges); */**/***Mansouramycin A (Marine *Streptomyces*); *cycloanthranilylproline (*Fuligo candida*); *Sannanine (*Streptomyces sannanensis*); */**/***Caerulomycin F (Marine-derived *Acti,oalloteichus cyanogriseus*); ***(2-aminophenyl)(2-ethyloxazol-5-yl)methanone (*Streptomyces* sp.)	+	+	+	+	+	+	23, 50-59
3.80	**231.1147**	**$[M+H]^+$**	231.1134	$C_{13}H_{14}N_2O_2$	4.5	***(3*S*,8a*S*)-3-Phenyloctahydropyrrolo[1,2-a]pyrazine-1,4-dione (*Penicillium cluniae* Quintanilla (CECT 2888), *Cyclo(Phe-Dehydroabu) (*Streptomyces* sp. Goe 40/14); Tetrahydro-1-methyl-β-carboline carboxylic acid (*Amanita muscaria*)	-	-	+	-	-	-	41, 60, 61
4.06	**130.1229**	**$[M+H]^+$**	130.1232	$C_7H_{15}NO$	2.5	*Isopentyl acetamide (Marine bacteria)	-	-	+	-	+	+	62
4.08	**171.1502**	**$[M+H]^+$**	171.1497	$C_9H_{18}N_2O$	2.6	no hit in antibase/DNP/Reaxys	-	-	+	-	+	-	
4.49	**164.1077**	**$[M+H]^+$**	164.1075	$C_{10}H_{13}NO$	1.2	*3-Acetamide derivatives; */***Streptazone D (*Streptomyces* FORM5 and *Streptomyces* A1); */**Dihydroabikoviromycin (*Streptomyces olivaceus, S. vinidochromogenes, S. reticuli, S. anulatus*); ***Methyl-*N*-(benzyl-methyl)-formamide (*Actinomadura* sp.; cultures of limnic strains GW102a and GW73a)	+	+	+	+	+	+	20, 36, 63-66
5.70	**437.2023**	**$[M+Na]^+$**	437.1999	$C_{17}H_{34}O_{11}$	3.9	no hit in antibase/DNP/Reaxys	-	+	-	-	-	-	
6.16	**255.0666**	**$[M+H]^+$**	255.0657	$C_{15}H_{10}O_4$	3.4	**1,8-Dihydroxy-4-methylanthraquinone (Cyanobacteria); */***Chrysophanol (*Penicillium* sp.); *Daidzein (*Micromonospora halophytica*, *Streptomyces* sp.)	-	+	+	+	+	+	67-69
6.34	**268.2031**	**$[M+H]^+$**	268.2025	$C_{14}H_{25}N_3O_2$	2.1	no hit in antibase/DNP/Reaxys	+	-	+	-	+	-	
6.61	**303.1711**	**$[M+H]^+$**	303.1709	$C_{17}H_{22}N_2O_3$	0.8	*/**/***Trichostatin (*Streptomyces hygroscopicus* a-300 (FERM-p 1312), *Streptomyces* sp. RK98-A74) ; ***Brocaeloid B (*Penicillium brocae* MA-192), **Terretrione B (mangrove-derived *Aspergillus terreus* A8-4)	-	+	-	-	-	-	70-73
6.74	**280.1240**	**$[M+H]^+$**	280.1232	$C_{12}H_{17}N_5OS$	2.8	no hit in antibase/DNP/Reaxys	+	+	+	+	-	+	
6.81	**544.2379**	**$[M+Na]^+$**	544.2370	$C_{23}H_{39}NO_{12}$	1.7	no hit in antibase/DNP/Reaxys	-	-	-	-	+	-	
6.83	**504.2800**	**$[M+Na]^+$**	504.2785	$C_{22}H_{43}NO_{10}$	3.1	no hit in antibase/DNP/Reaxys	-	-	-	-	+	-	

6.89	**341.1490**	**$[M+Na]^+$**	341.1477	$C_{17}H_{22}N_2O_4$	3.8	*/**Phomamide (*Phoma lingam*) ; */**Tomaymicin (*Nocardia* sp. C-15003, *Streptomyces achromogenes* var. *tomaymyceticus*) ; */**Terragine B (DNA isolated from soil expressed in a *Streptomyces lividans* host) ; */**Daryamide C (marine-derived *Streptomyces* strain CNQ-085) ; */**Fusaperazine C (endophytic *Colletotrichum gloeosporioides*, *Penicillium crustosum*, *Fusarium* sp.)	-	+	-	-	-	-	74-80
7.75	**513.2674**	**$[M+H]^+$**	513.2673	$C_{22}H_{36}N_6O_8$	0.1	no hit in antibase/DNP/Reaxys	+	-	-	-	-	-	
8.71	**670.3077**	**$[M+Na]^+$**	n.d.	Multiple choices	n.d.	n.d.	-	-	-	-	+	-	
8.94	**247.1322**	**$[M+Na]^+$**	247.1310	$C_{13}H_{20}O_3$	4.7	*/**Hyalopyrone (*Ascochyta hyalospora, Sporormiella teretispora* and marine-derived *Ascochyta salicorniae*) ; */**Similin-A (*Sporormiella similis*) ; *5-(6'-Methyl-7'-oxo-octyl)-(5*H*)-furan-2-one or MKN-003A (marine *Streptomyces* sp. B 3497, B8042) ; */**11-Hydroxy-4-methyl-2,4,6-dodecatrienoic acid (*Mucor* sp. KL 94-42) ; */**Guaymasol and epiguaymasol (marine deep-sea *Bacillus*) ; */**4,5-Didehydroacaterin (*Pseudomonas* sp. A92 and *P. jessenii*) ; */**Apiosporic acid (marine endophytic *Apiospora montagnei*) ; */**2,4-Dimethoxy-6-pentylphenol (endophytic fungus *Botryosphaeria mamane* PSU-M76) ; **N*-[5-(2-Formyl-5-hydroxymethyl-pyrrol-1-yl)-pentyl]-acetamide (endophytic *Fusarium incarnatum* (HKI00504) from mangrove) ; */**Cyclopenicillone (*Penicillium decumbens*); ** Petrynol (*Petrosia* sp.); ** Mollipilin D (*Chaetomium mollipilium*); **3-Hydroxy-5-magastigmene-7,9-dione (*Prorocentrum minimum*)	-	-	+	-	-	-	81-94
8.95	**288.1585**	**$[M+H]^+$**	288.1600	$C_{17}H_{21}NO_3$	4.7	*/**/***Trichostatic acid (*Streptomyces sioyaensis*; *Streptomyces* sp.), *3-(2'-Acetoxy-3'-oxo-4'-methylhexyl)-indole (bacterial symbiont *Xenorhabdus bovienii* A2), *Solanapyrone G (marine filamentous fungus)	-	-	+	-	-	-	95-98
9.41	**624.3024**	**$[M+Na]^+$**	n.d.	Multiple choices	n.d.	n.d.	-	-	-	-	+	-	
9.69	**668.2924**	**$[M+Na]^+$**	n.d.	Multiple choices	n.d.	n.d.	-	-	-	-	+	-	
10.00	**654.3116**	**$[M+Na]^+$**	n.d.	Multiple choices	n.d.	n.d.	-	-	-	-	+	-	
15.10	**1013.4707**	**$[M+Na]^+$**	n.d.	Multiple choices	n.d.	*** New compound based on high resolution mass	-	-	-	-	+	-	
15.71	**205.1963**	**$[M+H]^+$**	205.1956	$C_{15}H_{24}$	3.3	Sesquiterpenes e.g. */**/*** pentalenene (*Streptomyces griseochromogenes*), *Bicyclogermacrene, δ-elemene, eepldozene, β-bourbonene (*S. griseus* CBS 109.60), *α-Gurjunene, kelsoene, bourbon-11-ene, cadina-3,5-diene, ε-cadinene, β-muurolene, cadina-1,4-diene, eudesma-5,11-diene (marine *Streptomyces* sp. GWS-BW-H5)	-	-	+	-	-	-	22, 99-101
16.44	**1279.5986**	**$[M+Na]^+$**	n.d.	Multiple choices	n.d.	n.d.	-	-	-	-	+	-	
17.29	**1293.6152**	**$[M+Na]^+$**	n.d.	Multiple choices	n.d.	n.d.	-	-	-	-	+	-	
17.37	**1271.6277**	**$[M+Na]^+$**	n.d.	Multiple choices	n.d.	n.d.	-	-	-	-	+	-	
17.64	**1263.6039**	**$[M+Na]^+$**	n.d.	Multiple choices	n.d.	n.d.	-	-	-	-	+	-	
18.32	**279.1614**	**$[M+H]^+$**	279.1596	$C_{16}H_{22}O_4$	3.1	**Coibanole A (*Pycnoporus sanguineus*); */**Oncorhyncolide (Marine bacterium); **Penicitrinol D (marine-derived *Penicillium citrinum*); **Guignardone D (endophytic fungus A1 from *Scyphiphora hydrophyllacea*); */*** (*R*)-de-*O*-Methyllasiodiplodin (endophytic fungus *Lasiodiplodia* sp.); *Isoacrostalidic acid (*Acrostalagmus* sp.); *1-(3,5-Dihydroxyphenyl)-10'-hydroxy-1-undecen-6'-one (*Gliocladium reseum*); *7-Oxo-brefeldin (*Penicillium* sp.); *Dechloromonilidiol (*Monilinia fructicola*); * Microsphaeropsisin (*Microphaeropsis* from marine sponges); *Cytosporone C (*Cytospora* sp.); *Hirsutic acid C (*Stereum hirsutum*); *Oblongolide G (*Phomopsis* sp.)	+	-	-	-	-	-	102-114
19.27	**1291.5997**	**$[M+Na]^+$**	n.d.	Multiple choices	n.d.	n.d.	-	-	-	-	+	-	
19.41	**1269.6169**	**$[M+H]^+$**	1269.6519	$C_{63}H_{88}N_{12}O_{16}$	24.2	*N*-Methyl-dactinomycin[3]	-	-	-	-	+	-	50, 115-120

19.56	**1255.6405**	**[M+H]$^+$**	1255.6363	$C_{62}H_{86}N_{12}O_{16}$	3.3	*/**/***Actinomycin D (*Streptomyces* sp.); *Aurantin II (*Streptomyces aurantiacus*)	-	-	-	-	+	-	114-119
19.67	**1277.6223**	**[M+Na]$^+$**	n.d.	Multiple choices	n.d.	n.d.	-	-	-	-	+	-	
19.91	**283.2763**	**[M+H]$^+$**	283.2749	$C_{17}H_{34}N_2O$	4.8	no hit in antibase/DNP/Reaxys	-	+	-	-	-	-	
20.09	**1291.6429**	**[M+Na]$^+$**	n.d.	Multiple choices	n.d.	n.d.	-	-	-	-	+	-	
20.14	**1291.6298**	**[M+Na]$^+$**	n.d.	Multiple choices	n.d.	n.d.	-	-	-	-	+	-	
20.79	**317.2700**	**[M+H]$^+$**	317.2692	$C_{18}H_{36}O_4$	2.5	**/***2,3-Dihydroxypropylpentadecanoate, 1-*O*-(13-methyltetradecanoyl)-sn-glycerol (sponge *Stelletta* sp.), */*** Aggreceride A (*Streptomyces* strain om-3209; *(Streptomyces* sp.)	-	+	-	-	-	-	120, 121
21.35	**297.2905**	**[M+H]$^+$**	297.2906	$C_{18}H_{36}N_2O$	0.3	no hit in antibase/DNP/Reaxys	-	-	-	+	-	-	
22.64	**436.2484**	**[M+H]$^+$**	436.2479	$C_{22}H_{34}N_5O_2Cl$	1.2	no hit in antibase/DNP/Reaxys	-	+	-	-	-	-	
24.12	**434.3253**	**[M+H]$^+$**	434.3270	$C_{26}H_{43}NO_4$	4.1	no hit in antibase/DNP/Reaxys	-	-	+	-	-	-	
24.47	**226.0144**	**[M+H]$^+$**	226.0132	$C_7H_4N_5O_2Cl$	5.0	no hit in antibase/DNP/Reaxys	+	-	-	-	-	-	

1. Lacret, R. et al. New ikarugamycin derivatives with antifungal and antibacterial properties from *Streptomyces zhaozhouensis*. Mar. Drugs 13, 128–140 (2015).

2. Li, X., Zhou, Y., Zhu, R., Chang, W. & Yuan, H. Identification and Biological Evaluation of secondary Metabolites from the Endolichenic Fungus *Aspergillus versicolor*. Chem. Biodivers. 12,

3. Shaala, L. A. & Youssef, D. T. A. Identification and Bioactivity of Compounds from the Fungus *Penicillium* sp. CYE-87 Isolated from a Marine Tunicate. Mar. Drugs 13, 1698–1709 (2015).

4. Asiri, I. A. M., Badr, J. M. & Youssef, D. T. A. Penicillivinacine , antimigratory diketopiperazine alkaloid from the marine-derived fungus *Penicillium vinaceum*. Phytochem. Lett. 13, 53–58

5. Yonezawa, K., Yamada, K. & Kouno, I. New Diketopiperazine Derivatives Isolated from Sea Urchin-Derived *Bacillus* sp . Chem. Pharm.Bull 59, 106–108 (2011).

6. Veluri, R., Oka, I., Wagner-dobler, I. & Laatsch, H. New Indole Alkaloids from the North Sea Bacterium *Vibrio parahaemolyticus*. J. Nat. Prod. 66, 1520–1523 (2003).

7. Lewis, E. A., Adamek, T. L., Vining, L. C. & White, R. L. Metabolites of a Blocked Chloramphenicol Producer. J. Nat. Prod. 66, 62–66 (2003).

8. Ying, Y., Zhan, Z. & Ding, Z. Bioactive Metabolites From Penicillium sp . P-1 , A Fungal endophyte In *Huperzia serrata*. Chem. Nat. Compd. 47, 481–484 (2011).

9. Wu, S. J., Fotso, S., Li, F., Qin, S. & Laatsch, H. Amorphane Sesquiterpenes from a Marine *Streptomyces* sp . ⊥ , 1. J. Nat. Prod. 70, 304–306 (2007).

10. Schlorke, O. et al. Structure and Biosyntesis of cetoniacytone A, a cytotoxic aminocarba sugar produced by an endosymbiotic *Actinomyces*. J. Antibiot. (Tokyo). 55, 635–642 (2002).

11. Pusecker, K. & Laatsch, H. Dihydrophencomycin Methyl Ester , a New Phenazine Derivative from a Marine Streptomycete. J. Antibiot. (Tokyo). 50, 479–483 (1997).

12. Biabani, M. A. F., Helmke, E. M., Lovisetto, B., Laatsch, H. & Weyland, H. Anthranilamides: new antimicroalgal active substances from a marine *Streptomyces* sp. J. Antibiot. (Tokyo). 51, 333– (1997).

13. Wang, F., Xu, M., Li, Q., Sattler, I. & Lin, W. p-Aminoacetophenonic Acids Produced by a Mangrove Endophyte *Streptomyces* sp. (strain HK10552). Molecules 15, 2782–2790 (2010).

14. Shaaban, M., Maskey, R. P., Wagner-dobler, I. & Laatsch, H. Pharacine , a Natural p -Cyclophane and Other Indole Derivatives from *Cytophaga* sp . Strain AM13 . 1 1. J. Nat. Prod. 65, 1660–1663 (2002).

15. Chapla, V. M. et al. Antifungal Compounds Produced by *Colletotrichum gloeosporioides*, an Endophytic Fungus from *Michelia champaca*. Molecules 19, 19243–19252 (2014).

16. Tadahiko, H., Michihiko, K., Hiroshi, N., Takaaki, A. & Hamao, U. p--Hydroxyphenylacetaldoxime, an inhibitor of β-Galactosidase, produced by Actinomycetes. J. Antibiot. (Tokyo). XXXII, 91–93 (1979).

17. Jeong, S., Shin, H. J., Kim, T. S., Lee, H. & Park, S. Streptokordin , a New Cytotoxic Compound of the Methylpyridine Class from a Marine-derived *Streptomyces* sp . KORDI-3238. J. Antibiot. (Tokyo). 59, 234–240 (2006).

18. Rezanka, T., Libalova, D., Votruba, J. & Viden, I. Identification of odorous compounds from *Streptomyces avermitilis*. Biotechnol. Lett. 16, 75–78 (1994).

19. Maskey, R. P., Huth, F., Grun-Wollny, I. & Laatsch, H. 2-alkyl-3,4-dihydroxy-5-hydroxymethylpyridine derivatives: New Natural Vitamin B6 Analogues from a Terrestrial *Streptomyces* sp . Z. Naturforsch 60b, 63–66 (2005).

20. Dembitsky, V. M., Shkrob, I. & Lev, O. Occurrence of Volatile Nitrogen-Containing. J. Chem. Ecol. 26, 1359–1366 (2000).

21. Maskey, R. P., Shaaban, M., Grun-Wollny, I. & Laatsch, H. Quinazolin-4-one Derivatives from *Streptomyces* Isolates. J. Nat. Prod. 67, 1131–1134 (2004).

22. Dickschat, J. S., Martens, T., Brinkhoff, T., Simon, M. & Schulz, S. Volatiles Released by a *Streptomyces* Species Isolated from the North Sea. Chem. Biodivers. 2, 837–865 (2005).

23. Kornsakulkarn, J., Saepua, S., Supothina, S., Chanthaket, R. & Thongpanchang, C. Sporaridin and sporazepin from actinomycete *Streptosporangium*. Phytochem. Lett. 10, 149–151 (2014).

24. Frohwein, Y. Z., Dafni, Z. & Friedman, M. New Metabolites of *Streptomyces alboniger*. Agric. Biol. Chem. 37, 679–680 (1973).

25. Sakai, S. et al. Elucidation of the structure of olivoretin A and D (Teleocidin B). Chem. Pharm.Bull 32, 354–357 (1984).

26. Farrell, L. W., Thaller, V. & Turner, J. L. Natural Acetylenes. Part 52. Polyacetylenic Acids and Aromatic Aldehydes from cultures of the fungus *Camarophyllus virgineus* (Wulfen ex Fr.) Kummer. J.C.S. Pekin I 1886–1888 (1977).

27. Fushimi, K., Anzai, K., Tokuyama, S., Kiriiwa, Y. & Matsumoto, N. Agrocybynes A e E from the culture broth of *Agrocybe praecox*. Tetrahedron 68, 1262–1265 (2012).

28. Dillman, R. L. & Cardellina II, J. H. Aromatic secondary metabolites from the sponge *Tedania ignis*. J. Nat. Prod. 54, 1056–1061 (1991).

29. Gross, B., Gallois, A., Spinnler, H. & Langlois, D. Volatile compounds produced by the ligninolytic fungus *Phlebia radiata* Fr . (Basidiomycetes) and influence of the strain specificity on the odorous profile. J. Biotechnol. 10, 303–308 (1989).

30. Ciolfi, G., Garofolo, A. & Di Stefano, R. Identification of some o-aminophenones as secondary metabolites of *Saccharomyces cerevisiae*. Vitis 34, 195–196 (1995).

31. Inoue, M. et al. Self-germination inhibitors from *Colletotrichum fragariae*. J. Chem. Ecol. 22, 2111–2122 (1996).

32. Omura, S. et al. Production of quinoline-2-methanol and quinoline-26methanol acetate by a new species of *Kitasatoa*, *Kitasatoa griseophaeus*. J. Antibiot. (Tokyo). XXIX, 797–803 (1976).

33. Böhlendorf, B. et al. Indole and quinoline derivatives as metabolites of tryptophan in Myxobacteria. Liebigs Ann. 49–53 (1996).

34. Yang, Z. et al. Sesquiterpenes from the Secondary Metabolites of *Streptomyces* sp . (YIM 56130). Chem. Pharm.Bull 59, 1430–1433 (2011).

35. Abdelfattah, M. S., Toume, K., Arai, M. a., Masu, H. & Ishibashi, M. Katorazone, a new yellow pigment with a 2-azaquinone-phenylhydrazone structure produced by *Streptomyces* sp. IFM 11299. Tetrahedron Lett. 53, 3346–3348 (2012).

36. Maskey, R. P., Asolkar, R. N., Kapaun, E., Wagner-Dobler, I. & Laatsch, H. Phytotoxic arylethylamides from Limnic bacteria using a screening with Microalgae. J. Antibiot. (Tokyo). 55, 643–649 (2002).

37. Lebuhn, M., Heulin, T. & Hartmann, A. Production of auxin and other indolic and phenolic compounds by *Paenibacillus polymyxa* strains isolated from different proximity to plant roots. FEMS Microbiol. Ecol. 22, 325–334 (1997).

38. Blum, S., Groth, I., Rohr, J. & Fiedler, H.-P. Biosynthetic capacities of actinomycetes. 5. Dioxolides, novel secondary metabolites from *Streptomyces tendae.* J. Basic Microbiol 36, 19–25

39. Keller-schierlein, W., Miiller, A., Hagmann, L., Schneider, U. & Zahner, H. (E)-3-(1H-Pyrrol-3-yl)-2-propensäure und (E)-3-(1H-Pyrrol-3-yl)-2-propensäureamid aus *Streptomyces parvulus,* Stamm Tü 2480. Helv. Chim. Acta 68, 559–562 (1985).

40. Pfeiffer, S., Bar, H. & Zarnack, J. Über Stoffwechselprodukte der Xanthocillin-bildende Mutante der *Penicillium notatum* Westl. Pharmazie1 27, 536–542 (1972).

41. Tumer, W. . Fungal Metabolites. (Academic Press, New York, 1971).

42. Jayatilake, G. S., Thornton, M, P., Leonard, A, L., Grimwade, J, E. & Baker, B, J. Metabolites from an Antarctic Sponge-Associated Bacterium, *Pseudomonas aeruginosa,* J. Nat. Prod., 59 (3), pp 293–296 (1996).

43. Rithner, C. D., Bunge, R. H., Bloem, R. J. & French, J. C. PD 125375, a novel metabolite co-produced with tomaymycin. J. Org. Chem 52, 298–300 (1987).

44. Gould, S. J. & Darling, S. Streptonigrin biosynthesis 2. Isolation of b-methyltryptophan and its intermediacy in the streptonigrin pathway. Tetrahedron 3207–3210 (1978).

45. Yang, S. & Cordell, G. A. Metabolism Studies of Indole Derivatives Using a Staurosporine Producer ,. J. Nat. Prod. 60, 44–48 (1997).

46. Popov, A. M. & Utkina, N. K. Pyrroloquinoline alkaloids from *Zyzzya* sp. sea sponges: isolation and antitumor activity characterization. Pharm. Chem. J. 32, 298–300 (1998).

47. Cho, J. Y. et al. Isolation and Structural Determination of the Antifouling Diketopiperazines from Marine-Derived *Streptomyces praecox* 291-11. Biosci. Biotechnol. Biochem. 76, 1116–1121 (2012).

48. Wang, F. et al. Seven new prenylated indole diketopiperazine alkaloids from holothurian- derived fungus *Aspergillus fumigatus*. Tetrahedron 64, 7986–7991 (2008).

49. Li, B., Chen, G., Bai, J., Jing, Y.-K. & Pei, Y.-H. A bisamide and four diketopiperazines from a marine-derived *Streptomyces* sp. J. Asian Nat. Prod. Res. 13, 1146–1150 (2011).

50. Parrot, D. Etudes de lichens marins, maritimes ou terrestres et des bactéries associées: Evaluation de la diversité et recherche de métabolites d'intérêts. (2014).

51. Davis, R. A. et al. Antimalarial Activity of Pyrroloiminoquinones from the Australian Marine Sponge *Zyzzya* sp. J. Med. Chem. 55, 5851–5858 (2012).

52. Dijoux, M.-G. et al. Antitumor activity and distribution of pyrroloiminoquinones in the sponge genus *Zyzzya*. Bioorg. Med. Chem. 13, 6035–6044 (2005).

53. Utkina, N. K., Makarchenko, A. E. & Denisenko, V. A. Zyzzyanones B - D, Dipyrroloquinones from the Marine Sponge *Zyzzya fuliginosa*. J. Nat. Prod. 68, 1424–1427 (2005).

54. Martínez-Luis, S., Gómez, J. F., Spadafora, C., Guzmán, H. M. & Gutiérrez, M. Antitrypanosomal alkaloids from the marine bacterium *Bacillus pumilus*. Molecules 17, 11146–55 (2012).

55. Fu, P. et al. Cytotoxic Bipyridines from the Marine-Derived Actinomycete. J. Nat. Prod. 74, 1751–1756 (2011).

56. Hawas, U. W. et al. Mansouramycins A - D, Cytotoxic Isoquinolinequinones from a Marine Streptomycete. J. Nat. Prod. 72, 2120–2124 (2009).

57. Raju, R., Gromyko, O., Fedorenko, V., Luzhetskyy, A. & Müller, R. Pimprinols A – C, from the terrestrial actinomycete, *Streptomyces* sp . Tetrahedron Lett. 53, 3009–3011 (2012).

58. Dembitsky, V. M., Rezanka, T., Spizek, J. & Hanus, L. O. Secondary metabolites of slime molds (myxomycetes). Phytochemistry 66, 747–769 (2005).

59. Li, Y. et al. Sannanine, a new cytotoxic alkaloid from *Streptomyces sannanensis.* J. Antibiot. (Tokyo). 62, 647–648 (2009).

60. Lopez-Gresa, P. M. et al. Insecticidal Activity of Paraherquamides, Including Paraherquamide H and Paraherquamide I, Two New Alkaloids Isolated from *Penicillium cluniae*. J. Agric. Food Chem. 54, 2921–2925 (2006).

61. Bode, H. B., Bethe, B., Höfs, R. & Zeeck, A. Big Effects from Small Changes: Possible Ways to Explore Nature's Chemical Diversity. ChemBioChem 3, 619–627 (2002).

62. Schulz, S. et al. Biological Activity of Volatiles from Marine and Terrestrial Bacteria. Mar. Drugs 8, 2976–2987 (2010).

63. Puder, C., Krastel, P. & Zeeck, A. Streptazones A, B1, B2, C, and D : New Piperidine Alkaloids from Streptomycetes. J. Nat. Prod. 1258–1260 (2000).

64. Maruyama, H. et al. Isoaltion of Abikoviromycin and dihydroabikoviromycin as inhibitors of polyketide synthase involved in melanin biosynthesis by *Colletotrichum lagenarium.* J. Antibiot. (Tokyo). 56, 801–804 (2003).

65. Kornsakulkarn, J., Saepua, S., Boonruangprapa, T., Suphothina, S. & Thongpanchang, C. New b-carboline and indole alkaloids from Actinomycete *Actinomadura* sp. BCC 24717.

66. Daoud, N. & Foster, H. Antifungal activity of Myxococcus species 1 production, Physicochemical and biological properties of antibiotics from *Myxococcus fulvus* S110 (Myxobacterales). Microbios 73, 173–184 (1993).

67. Wang, A., Wu, C. & Biehl, E. Unambiguous synthesis and spectral characterization of 1 , 8- dihydroxy-4-methylanthraquinone. ARKAT USA 80–84 (2002).

68. Gutierrez-Lugo, M. et al. Isolation of three new naturally occuring compounds from the culture of *Micromonospora* sp. P1068. Nat Prod Res. 19, 645–652 (2005).

69. Li, J. et al. Meroterpenes and azaphilones from marine mangrove endophytic fungus *Penicillium* 303#. Fitoterapia 97, 241–246 (2014).

70. Ueki, M. et al. A NewTrichostatin Derivative , Trichostatin RK, from *Streptomyces* sp. RK98-A74. J. Antibiot. (Tokyo). 54, 1093–1095 (2001).

71. Zhang, P. et al. Brocaeloids A – C , 4-Oxoquinoline and Indole Alkaloids with C-2 Reversed Prenylation from the Mangrove-Derived Endophytic Fungus *Penicillium brocae*. EurJoc 4029–4036 (2014). doi:10.1002/ejoc.201400067

72. Shen, Y. et al. Butyrolactone and Cycloheptanetrione from Mangrove-Associated Fungus *Aspergillus terreus.* Chem. Pharm.Bull 60, 1437–1441 (2012).

73. Leblond, B. & Beausoleil, E. Tricyclic hydroxamate and benzaminde derivatives, compositions and methods. (2005).

74. Soledade, M., Pedras, C., Séguin-Swartz, G., Abrams, S, R. Minor phytotoxins from the blackleg fungus *Phoma lingam*. Phytochemistry, 29(3), 777-782 (1990).

75. Kariyone, K., Yazawa, H., Kohsaka, M. The Structures of Tomaymycin and Oxotomaymycin, Chemical and Pharmaceutical Bulletin 19(11), 2289-2293 (1971).

76. Arima, K., Kohsaka, M., Tamura, G., Imanaka, H., Sakai, H. Studies on tomaymycin, a new antibiotic. I isolation and properties of tomaymyin, The Journal of Antibiotics 25(8), 437-444 (1972).

77. Kaneko, T.,Wong, H, S, L. Patent, Process for conversion of oxotomaymycin to tomaymycin US4,427,588 Jan. 24, (1984)

78. Wang, G-Y-S. et al., Novel Natural Products from Soil DNA Libraries in a Streptomycete Host, Org. Lett., 2(16), 2401–2404 (2000).

79. Asolkar R, N., Jensen P, R., Kauffman C, A., Fenical W. Daryamides A-C, weakly cytotoxic polyketides from a marine-derived actinomycete of the genus *Streptomyces* strain CNQ-085, J Nat Prod. 69(12), 1756-9 (2006).

80. Guimarães, D, O. et al., Diketopiperazines produced by endophytic fungi found in association with two Asteraceae species, Phytochemistry, 71(11–12), 1423-1429 (2010).

81. Venkatasubbaiah, P., Chilton, W, S. Phytotoxins of *Ascochytahyalospora*, Causal Agent of Lambsquarters Leaf Spot, J. Nat. Prod., 55(4), 461–467 (1992).

82. Simon F. et al., Polyketides from the marine-derived fungus *Ascochyta salicorniae* and their potential to inhibit protein phosphatases, Org. Biomol. Chem., 4, 2233-2240 (2006).

83. Weber, H, A., Swenson, D, C., Gloer, J, B. Similins A and B: New antifungal metabolites from the coprophilous fungus *Sporormiella similis*,Tetrahedron Lett., 33, 1157-60 (1992).

84. Mukku, V. J. R. V., Speitling, M., Laatsch, H., Helmke, E. New Butenolides from Two Marine Streptomycete, J. Nat. Prod., 63(11), 1570–1572 (2000).

85. Lorenzen, K., Anke, T., Sterner, O. 11-Hydroxy-4-methyl-2,4,6-dodecatrienoic acid from fermentations of a *Mucor* species, Phytochemistry, 43(4), 791–792 (1996).

86. Trischman, J, A., Jensen, P, R., Fenical, W. Aromatic Triols from a Deep-Sea *Bacillus* Isolate, Natural Product Letters, 11(4), 279-284 (1998).

87. Sekiyama, Y., Araya, H., Hasumi, K., Endo, A., Fujimoto, Y. Biosynthesis of acaterin: Incorporation of glycerol into the C 3 branched unit, Tetrahedron Lett., 39, 6233(1998).

88. Klemke, C., Kehraus, S., Wright A, D., König G, M., New secondary metabolites from the marine endophytic fungus *Apiospora montagnei*, J Nat Prod., 67(6), 1058-63 (2004).

89. Pongcharoen, W., Rukachaisirikul, V., Phongpaichit, S., Sakayaroj, J. A New Dihydrobenzofuran Derivative from the Endophytic Fungus *Botryosphaeria mamane* PSU-M76 Chemical and Pharmaceutical Bulletin., 55(9), 1404-1405 (2007).

90. Li, L-Y. et al., Pyrrole and indole alkaloids from an endophytic *Fusarium incarnatum* (HKI00504) isolated from the mangrove plant *Aegicera scorniculatum*, Journal of Asian Natural Products Research., 10(8), 765-770 (2008).

91. Lin, S. et al., Cyclopenicillone, a unique cyclopentenone from the cultures of *Penicillium decumbens*, Chem. Commun., 47, 10413-10415 (2011).

92. Andersen, R, J.,Le Blanc, M, J., Sum, F, W. 1-(2,6,6-Trimethyl-4-hydroxycyclohexenyl)-1,3-butanedione, an extracellular metabolite from the dinoflagellate *Prorocentrum minimum*, J. Org. Chem., 45(6), 1169–1170 (1980).

93. Seo , Y., Cho, K, W., Lee, H-S., Rho, J-R., Shin, J. New Acetylenic Enol Ethers of Glycerol from the Sponge *Petrosia* sp., J. Nat. Prod., 62(1), 122–126 (1999).

94. Asai, T. et al., Structural Diversity of New C13-Polyketides Produced by *Chaetomium mollipilium* Cultivated in the Presence of a NAD+-Dependent Histone Deacetylase Inhibitor, Org. Lett., 14(2), 5456–5459 (2012).

95. Morioka, H. et al. A New Differentiation Inducer of Friend Leukemia cells, Trichostatic acid. Agric. Biol. Chem. 5, 1365–1370 (1985).

96. Jenkins, K. M., Toske, S. G., Jensen, P. R. & Fenical, W. Solanapyrones E-G antialgal metabolites produced by a marine fungus. Phytochemistry 49, 2299–2304 (1998).

97. Li, J., Chen, G., Webster, J. M. & Czyzewska, E. Antimicrobial metabolites from a bacterial symbiont. J. Nat. Prod. 58, 1081–1086 (1995).

98. Argoudelis, A. ., Coats, J. . & Johnson, L. . Directed biosynthesis of new celestosaminide antibiotics by *Streptomyces caelestis*. J. Antibiot. (Tokyo). XXVII, 738–743 (1974).

99. Mehta, G. & Rao, K. S. General transannulation approach to angular triquinanes. Total syntheses of (+/-)-pentalenene and (+/-)-epi-pentalenene. J. Am. Chem. Soc 108, 8015–8021

100. Pollak, F. C. & Berger, R. G. Geosmin and Related Volatiles in Bioreactor-Cultured *Streptomyces citreus* CBS 109 . 60. Appl. Environ. Microbiol. 62, 1295–1299 (1996).

101. Chen, S. et al. b-Resorcylic acid derivatives with a-glucosidase inhibitory activity from *Lasiodiplodia* sp. ZJ-HQ1, an endophytic fungus in the medicinal plant *Acanthus ilicifolius.* Phytochem. Lett. 13, 141–146 (2015).

102. Needham, J., Andersen, R. J. & Kelly, M. T. Oncorhyncolide, a novel metabolite of a bacterium isolated from seawater. Tetrahedron Lett. 32, 315–318 (1991).

103. Sato, M. & Kakisawa, H. Structures of Three New C16 Terpenoids from an *Acrostdagmus* Fungus. J.C.S. Pekin I 2407–2413 (1976).

104. Sassa, T., Nukina, M., Sugiyama, T. & Yamashita, K. Monilidiols , Characteristic and Bioactive Metabolites of Benomyl-resistant Strains of *Monilinia fructicola*. Agric. Biol. Chem. 47, 449–451 (1983).

105. Brady, S. F., Wagenaar, M. M., Singh, M. P., Janso, J. E. & Clardy, J. The Cytosporones , New Octaketide Antibiotics Isolated from an Endophytic Fungus. Org. Lett. 2, 4043–4046 (2000).

106. Comer, F. W. & Mkarbru, F. The structure and chemistry of hirsutic acid. Tetrahedron 23, 4761–4768 (1967).

107. Lin, T. et al. Three New Oblongolides from Phomopsis sp. XZ-01, an Endophytic Fungus from *Camptotheca acuminate*. Molecules 16, 3351–3359 (2011).

108. Chen, L. et al. Citrinin Derivatives from the Marine-Derived Fungus *Penicillium citrinum*. Chem. Pharm.Bull 59, 515–517 (2011).

109. Risuwan, K. T. et al. Lactone Derivatives from the Marine-Derived Fungus *Penicillium* sp . PSU-F44. Chem. Pharm.Bull 57, 1100–1102 (2009).

110. Shao, C. et al. Microsphaeropsisin A, a new sesquiterpenoid isolated from the mangrove endophytic fungus (No. DZ39). Nat Prod Res. 23, 1579–1583 (2009).

111. El-Sharkawy, S. & Abul-Hajj, Y. Microbial cleavage of zearalenone. Xenobiotica 18, 365–371 (1988).

112. Zheng, B. et al. Two new meroterpenes from endophytic fungus A of *Cyphiphora hydrophyllacea*. J. Asian Nat. Prod. Res. 14, 776–779 (2012).

113. Hollstein, U. Actinomycin. Chemistry and Mechanism of Action. Chem. Rev. 74, 625–652 (1973).

114. Grafe, U. et al. Aurantimycins , New Depsipeptide Antibiotics from *Streptomyces aurantiacus* IMET43917 Production , Isolation , Structure Elucidation, and Biological Activity. J. Antibiot. (Tokyo). 48, 119–125 (1994).

115. Nam, J. Y. et al. Actinomycin D, C2 and VII, inhibitors of Grb2-SHC interaction produced by *Streptomyces*. Bioorg. Med. Chem. Lett. 8, 2001–2 (1998).

116. Parrot, D. et al. Cyaneodimycin, a Bioactive Compound Isolated from the Culture of *Streptomyces cyaneofuscatus* Associated with *Lichina confinis*, EurJoc., 23, 3977-3982 (2016).

117. Orlova, T., Sorokina, N. & Silaev, A. Effects of amino acids on biosynthesis of actinomycin D by *Act. olivobrunneus* var. Krass. Antibiotiki1 17, 409–413 (1972).

118. Orlova, T., Silaev, A., Zheltova, A. & Saburova, V. Biosynthesis of phenols by *Actinomyces*, producer of aurantin, an antibiotic from the actinomycin group. Antibiotiki 13, 719–723 (1968).

119. Holmalahti, J., Santa, H., Laatsch, H. & Wright, A. Von. Comparison of the lethal effects of different actinomycins on a repair-deficient strain of *Escherichia coli*. Mutat. Res. 33–38

120. Zhao, Q. et al. New Lysophosphatidylcholines and Monoglycerides from the Marine Sponge *Stelletta* sp . J. Nat. Prod. 66, 725–728 (2003).

121. Omura, S., Nakagawa, A., Fukamachi, N., Otoguro, K. & Kobayashi, B. Aggreceride, a new platelet aggregatio inhibitor from *Streptomyces*. J. Antibiot. (Tokyo). XXXIX, 1180– (1986).
